# Supplementary material for: fingeRNAt—A novel tool for high-throughput analysis of nucleic acid-ligand interactions
Source: PLoS Comput Biol. 2022 Jun 2;18(6):e1009783. doi: 10.1371/journal.pcbi.1009783 (PMC9197077; doi:10.1371/journal.pcbi.1009783)

S1 Text. Interaction statistics for seven chemical groups of ligands: alcohols and polyols, nucleosides and nucleoside derivatives, amino acids, amino sugars and aminoglycosides, aliphatic amines, heterocycles, and others.

For each chemical class of the ligands (cluster), the medoid structure of the small molecule is presented. Some chemical classes of ligands (e.g., amino acids) were initially clustered into two groups; in such cases, these compounds were merged into a single group, and two medoids are presented. Statistics of detected interactions include **(A)** the total number of interactions detected for RNA-ligand complexes and **(B)** the percentage of RNA-ligand complexes with at least one occurrence of a given interaction.

Amino acids

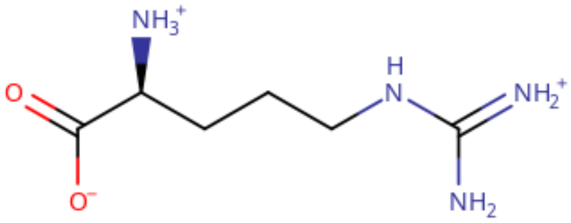

NH3+
O
O-
H
N
NH2+
NH2

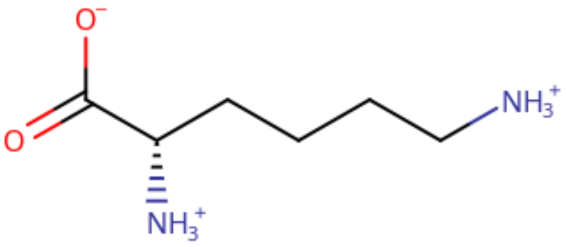

O-
O
NH3+
NH3+

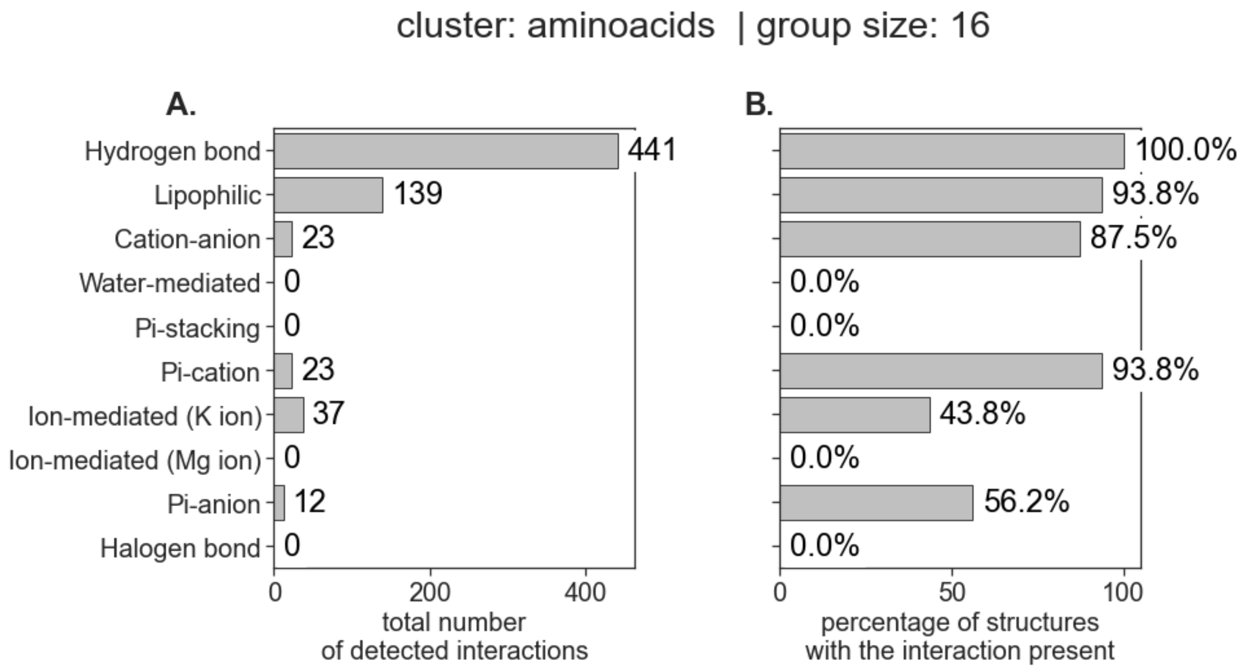

cluster: aminoacids | group size: 16
A.
Hydrogen bond
Lipophilic
Cation-anion
Water-mediated
Pi-stacking
Pi-cation
Ion-mediated (K ion)
Ion-mediated (Mg ion)
Pi-anion
Halogen bond
441
139
23
0
0
23
37
0
12
0
0
200
400
total number
of detected interactions
B.
100.0%
93.8%
87.5%
0.0%
0.0%
93.8%
43.8%
0.0%
56.2%
0.0%
0
50
100
percentage of structures
with the interaction present

Nucleosides and derivatives

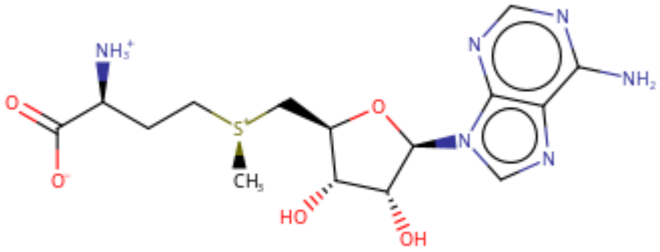

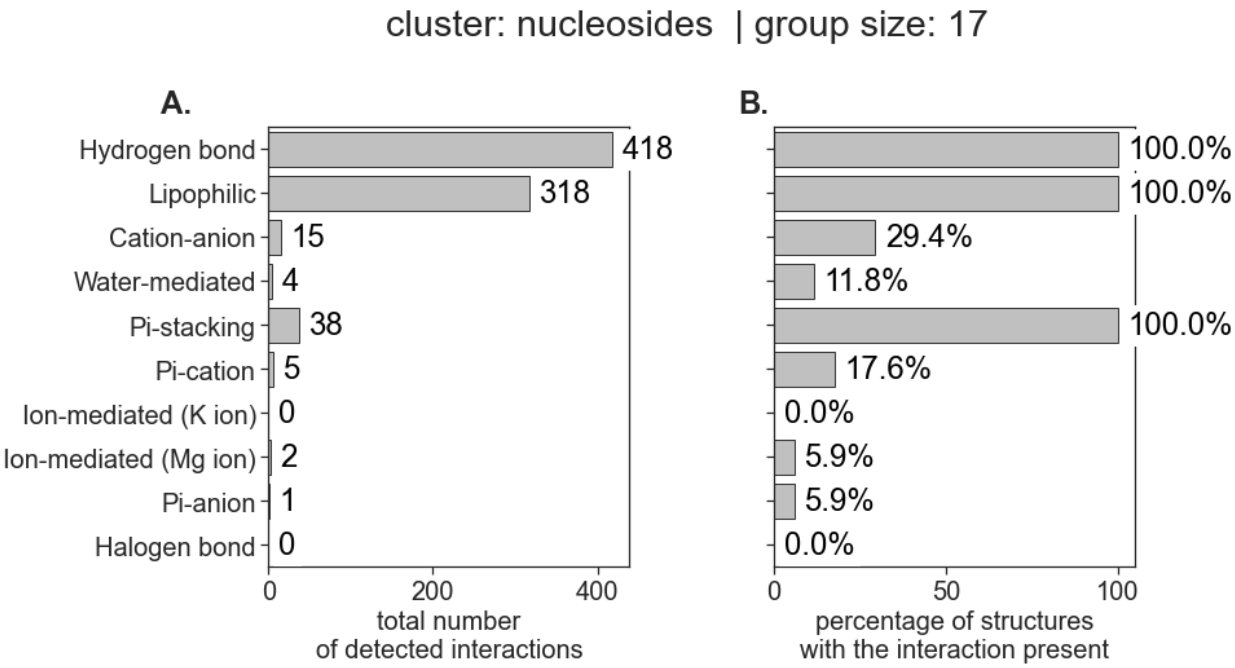

Amino sugars (including aminoglycosides)

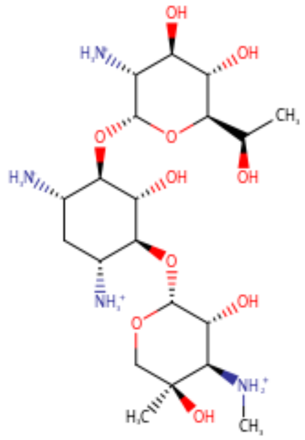

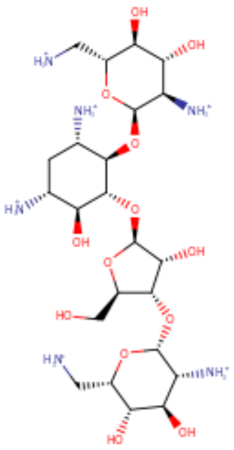

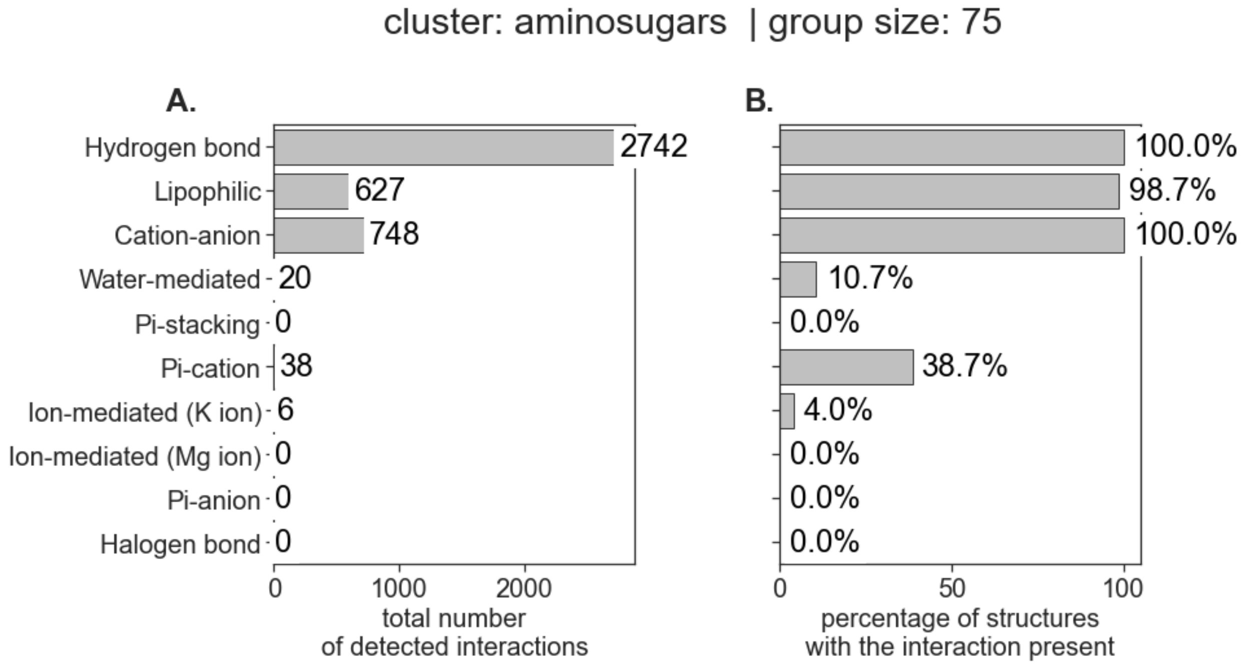

Heterocycles

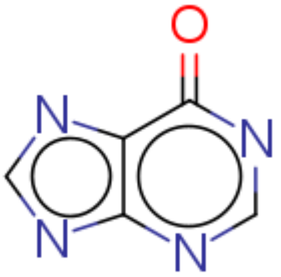

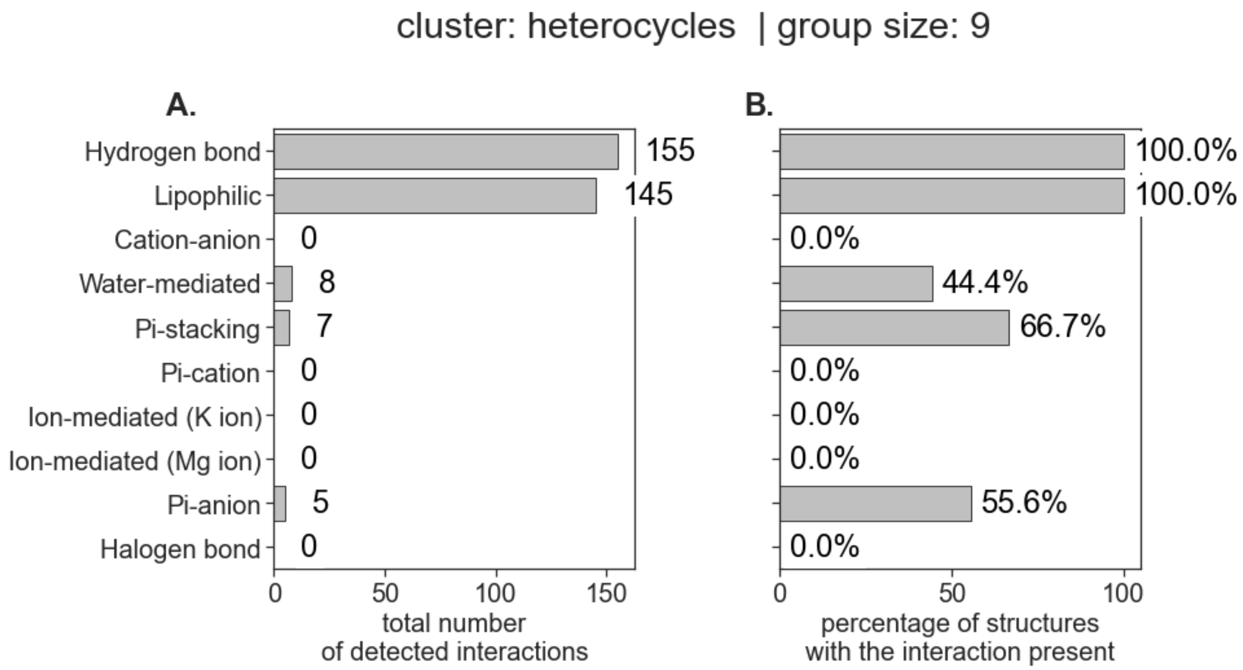

Aliphatic amines

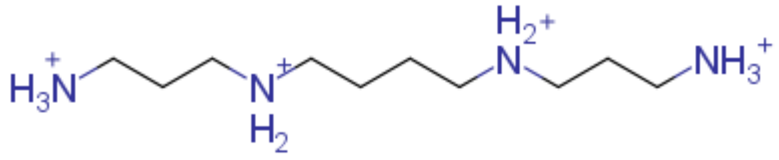

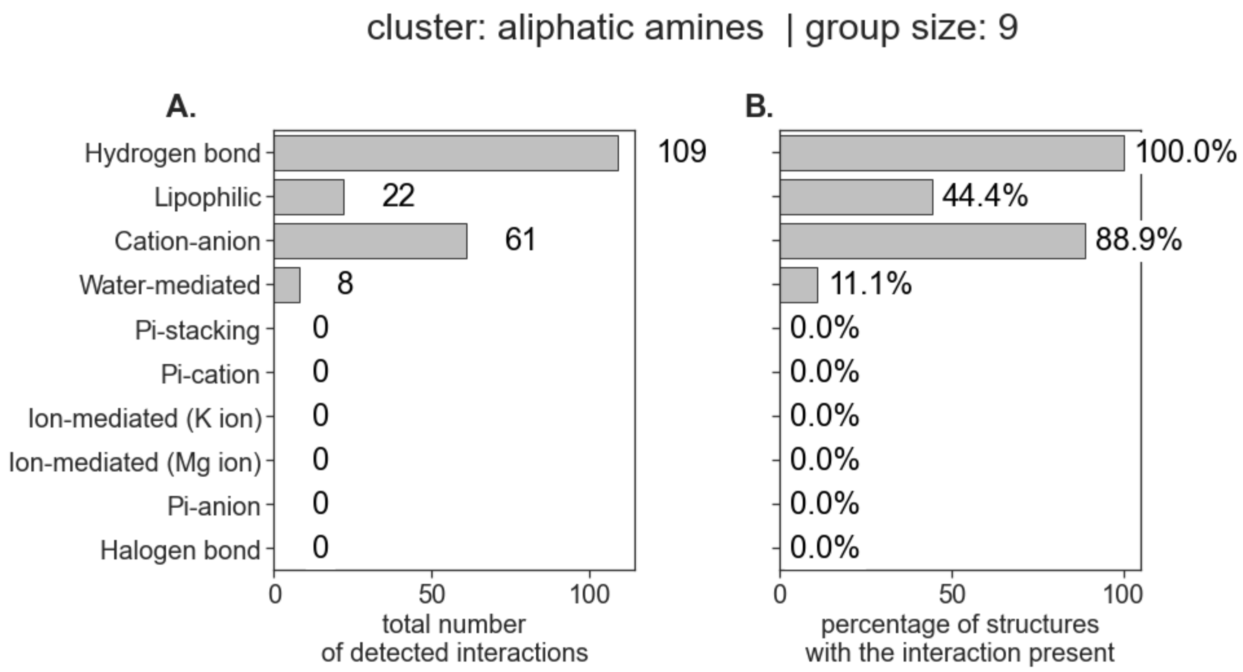

Alcohols and polyols

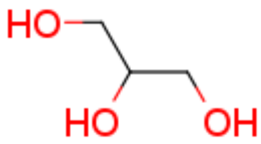

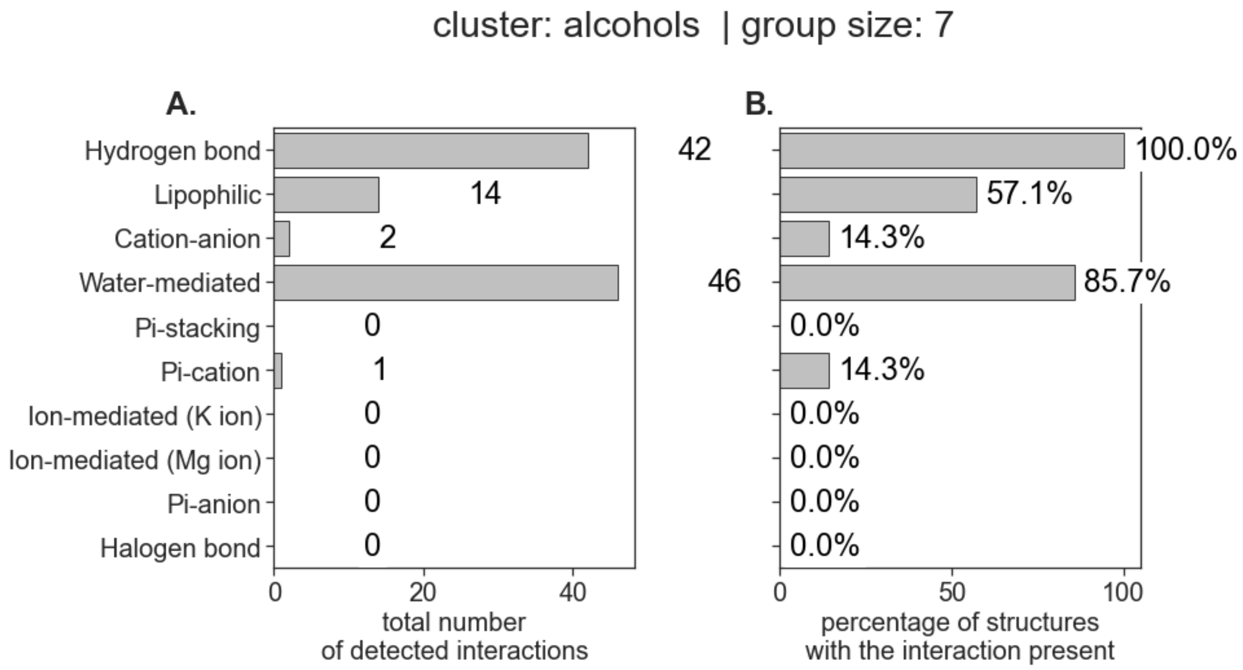

Other molecules

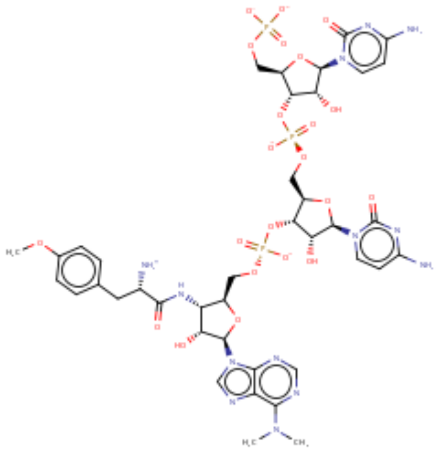

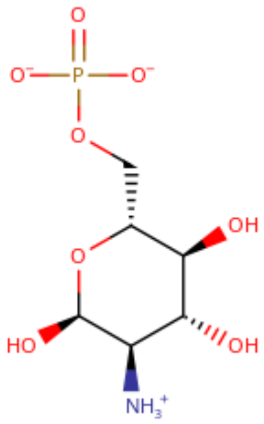

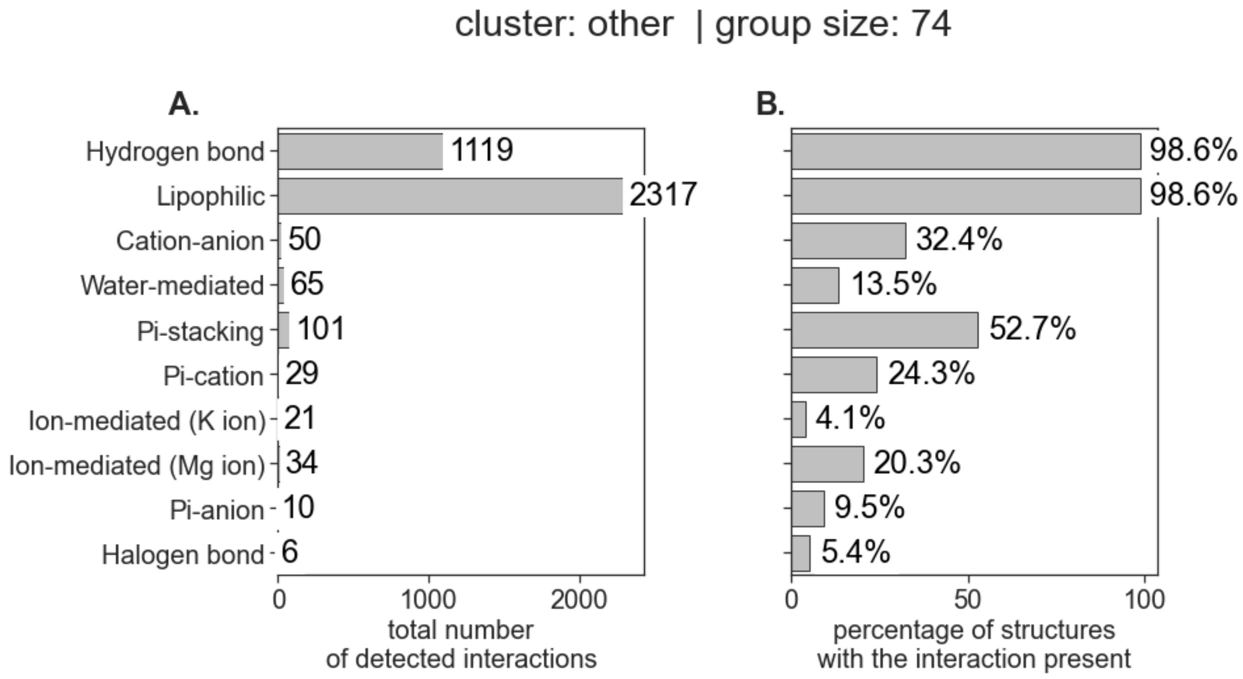

Supplement: S1 Text — (PDF) [file pcbi.1009783.s001.pdf]
