# Supplementary figures and images for: fingeRNAt—A novel tool for high-throughput analysis of nucleic acid-ligand interactions
Source: PLoS Comput Biol. 2022 Jun 2;18(6):e1009783. doi: 10.1371/journal.pcbi.1009783 (PMC9197077; doi:10.1371/journal.pcbi.1009783)

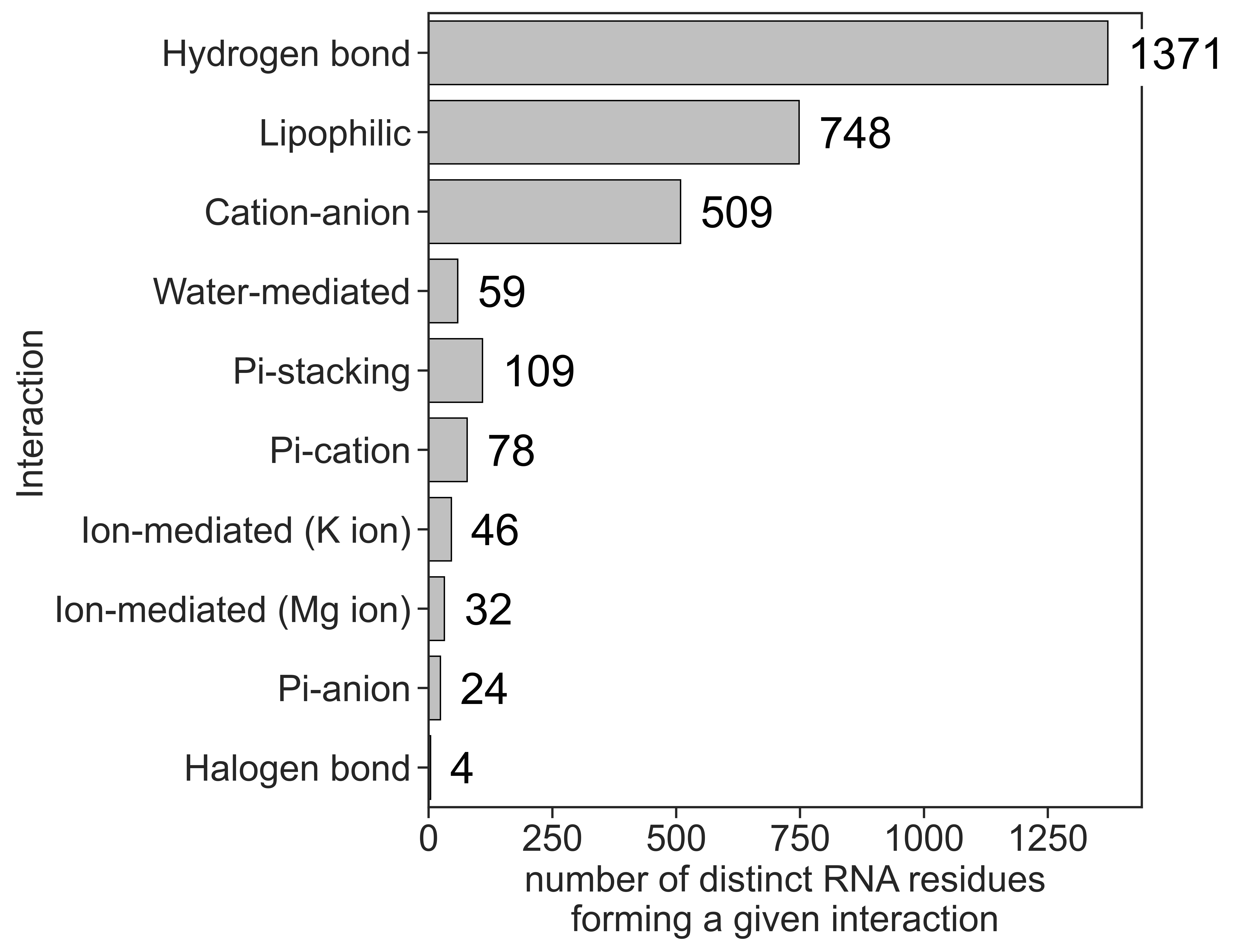

Supplement: S1 Fig — Counts for Pb, Mn, and Sr cations, which were present only in a single complex each, were removed for clarity. (PNG) [file pcbi.1009783.s002.png]

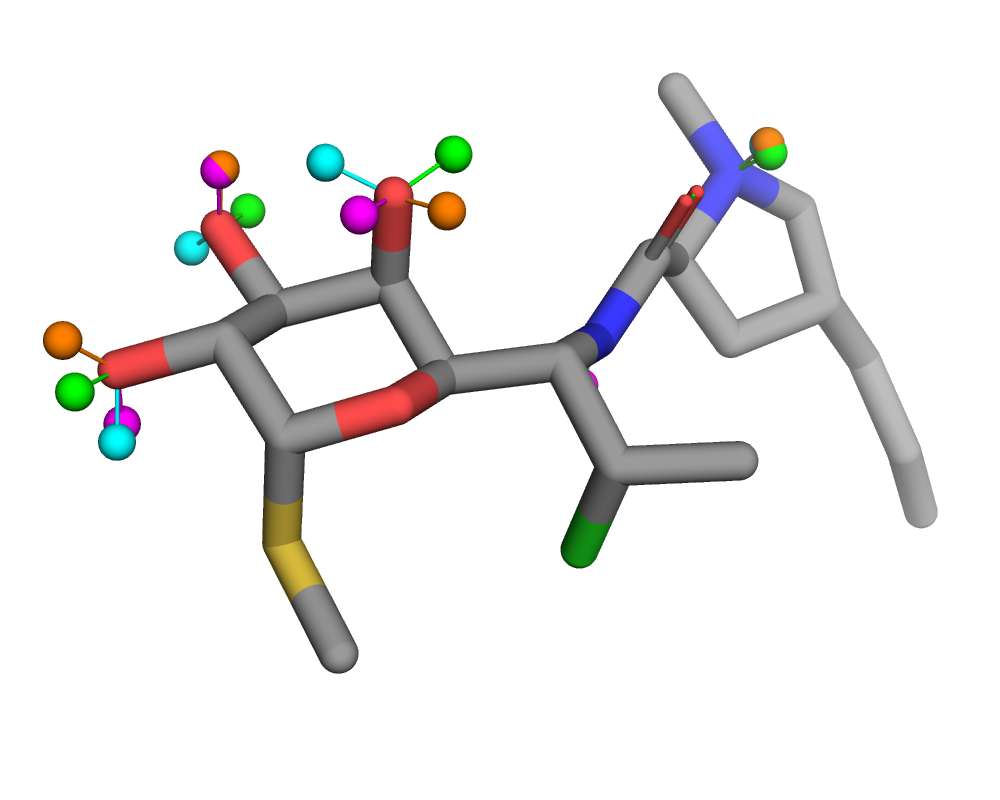

Supplement: S2 Fig — (PNG) [file pcbi.1009783.s003.png]

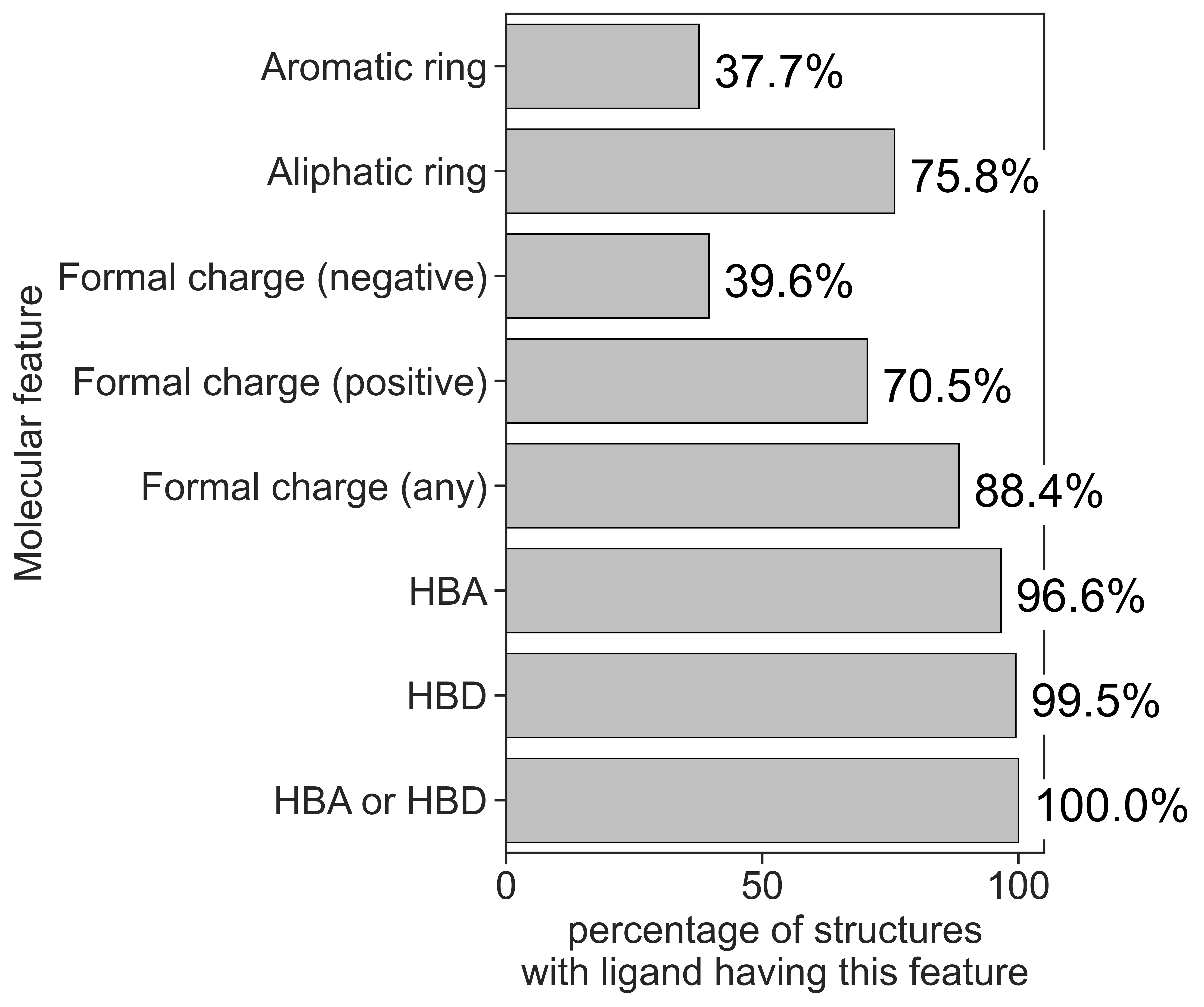

Supplement: S3 Fig — HBA—hydrogen bond acceptor; HBD—hydrogen bond donor. (PNG) [file pcbi.1009783.s004.png]

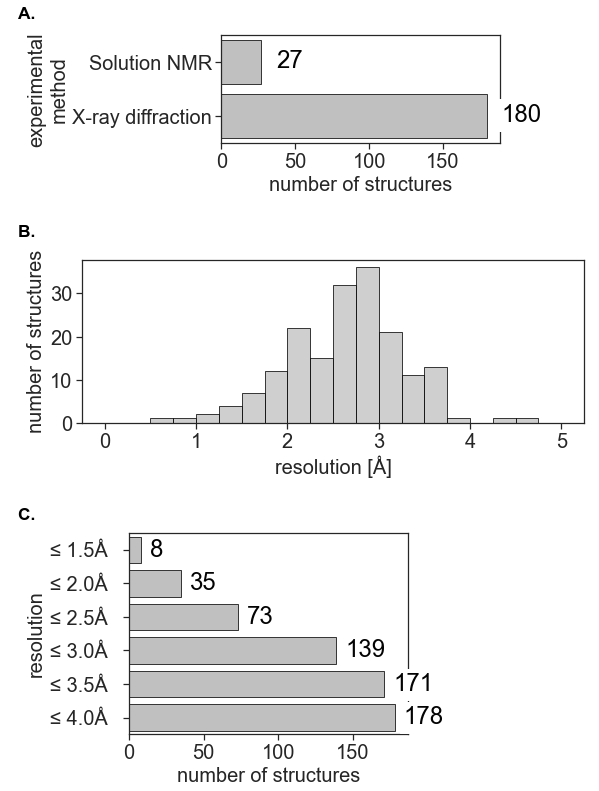

Supplement: S5 Fig — Statistics of the structures in the analyzed dataset: (A) structures count depending on the experimental method used; (B) resolution histogram for the structures determined by the X-ray diffraction (0.25 Å bin size); (C) number of structures with resolution below the given threshold. (PNG) [file pcbi.1009783.s006.png]

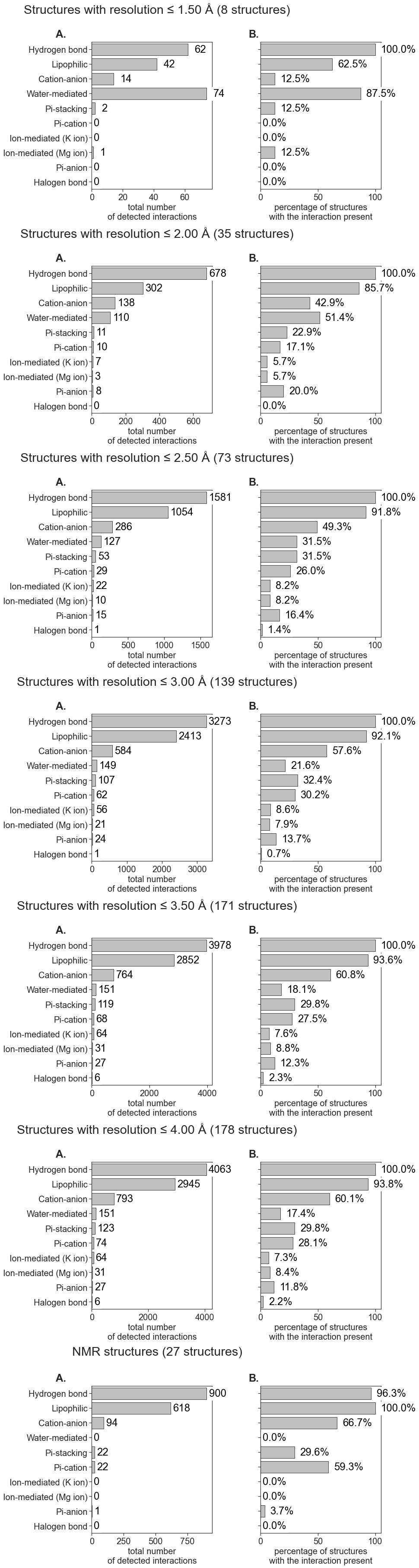

Supplement: S6 Fig — (A) Total number of interactions detected for RNA-ligand complexes; (B) the percentage of RNA-ligand complexes with at least one occurrence of a given interaction. (PNG) [file pcbi.1009783.s007.png]

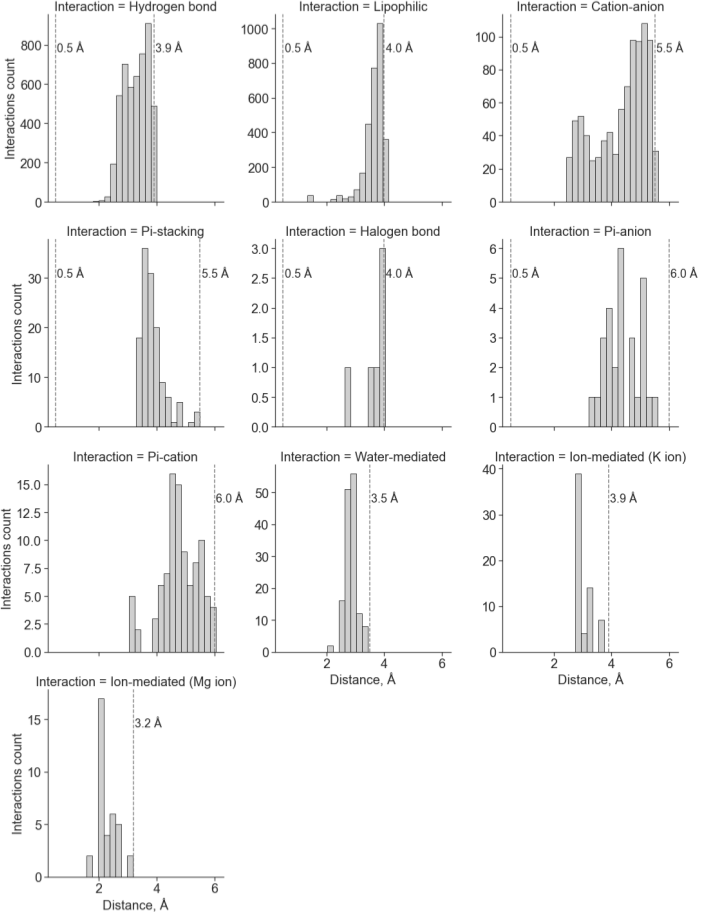

Supplement: S7 Fig — The minimum and maximum cut-off values for each interaction are marked with gray dashed lines. Histogram bin width is set to 0.2 Å. (PNG) [file pcbi.1009783.s008.png]

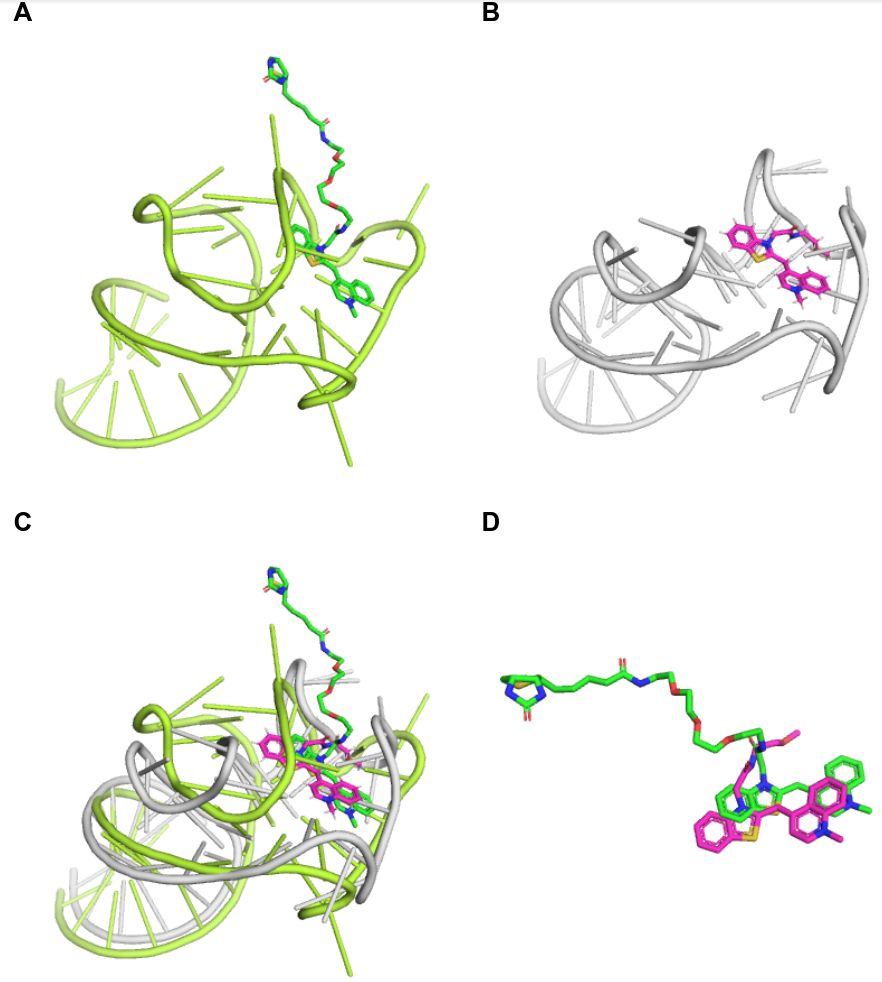

Supplement: S8 Fig — (A) The RNA-Puzzles round 23 solution and (B) the model submitted by the Das group (Das_7); (C) both complexes overlayed and (D) ligands. (PNG) [file pcbi.1009783.s009.png]

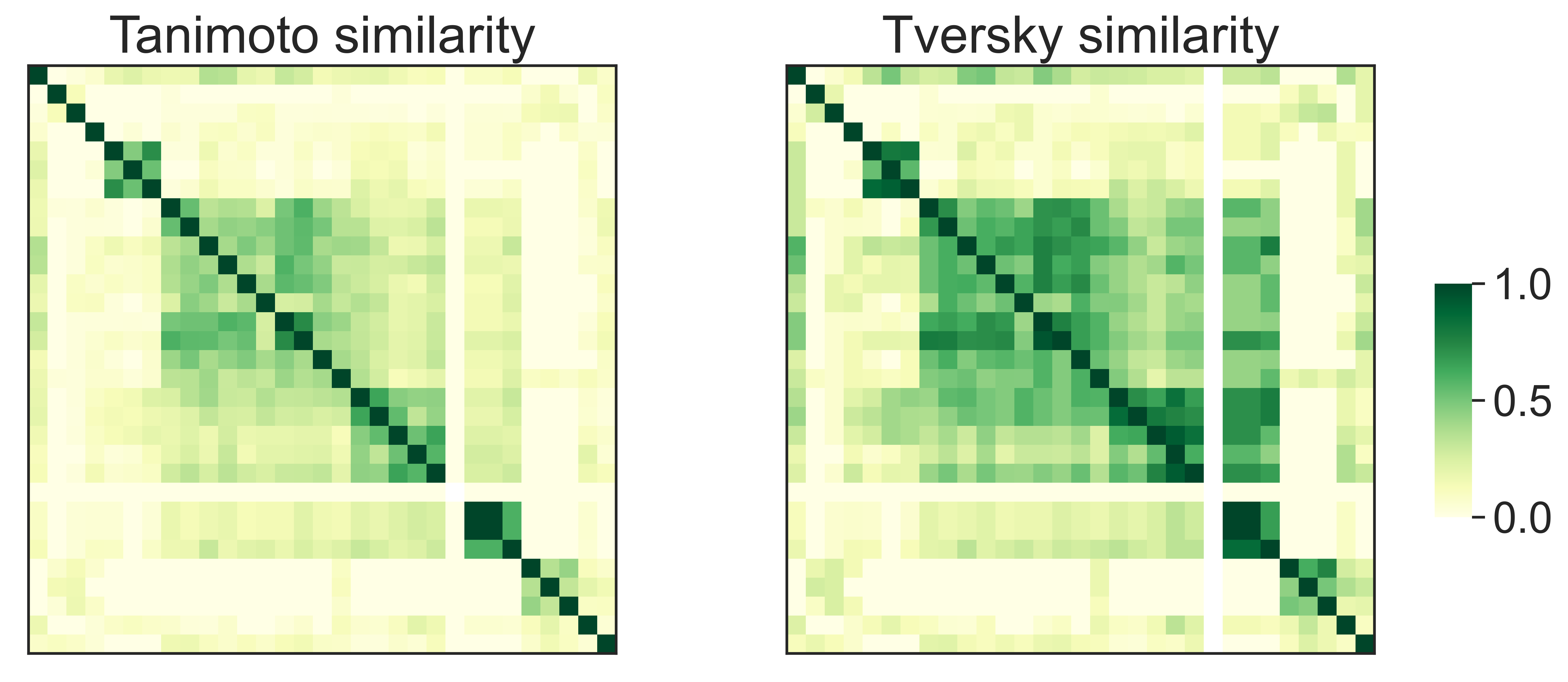

Supplement: S9 Fig — (PNG) [file pcbi.1009783.s010.png]

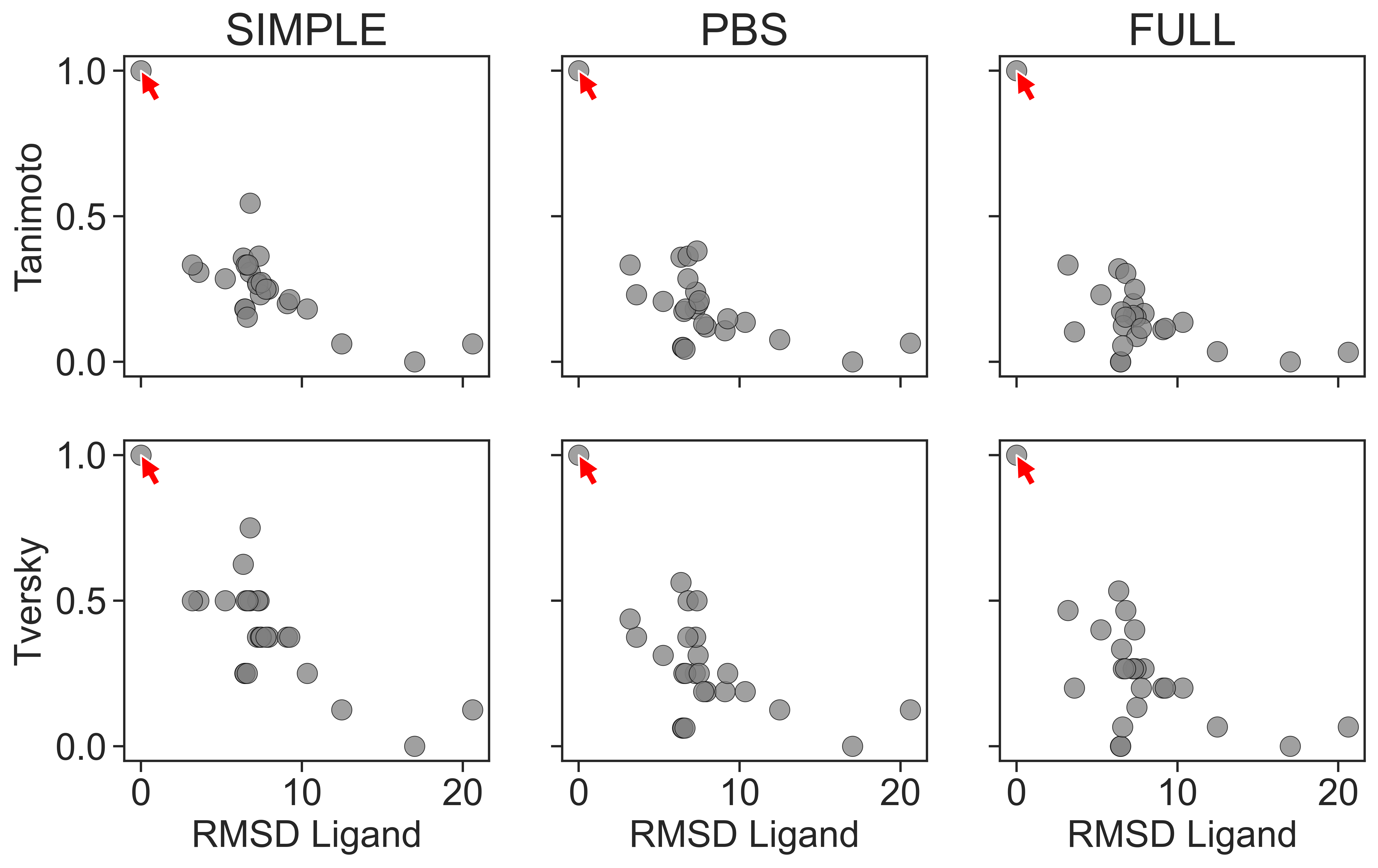

Supplement: S11 Fig — Ligand RMSD was calculated for the TO1 N-acetamide substructure common in all submitted models. (PNG) [file pcbi.1009783.s012.png]

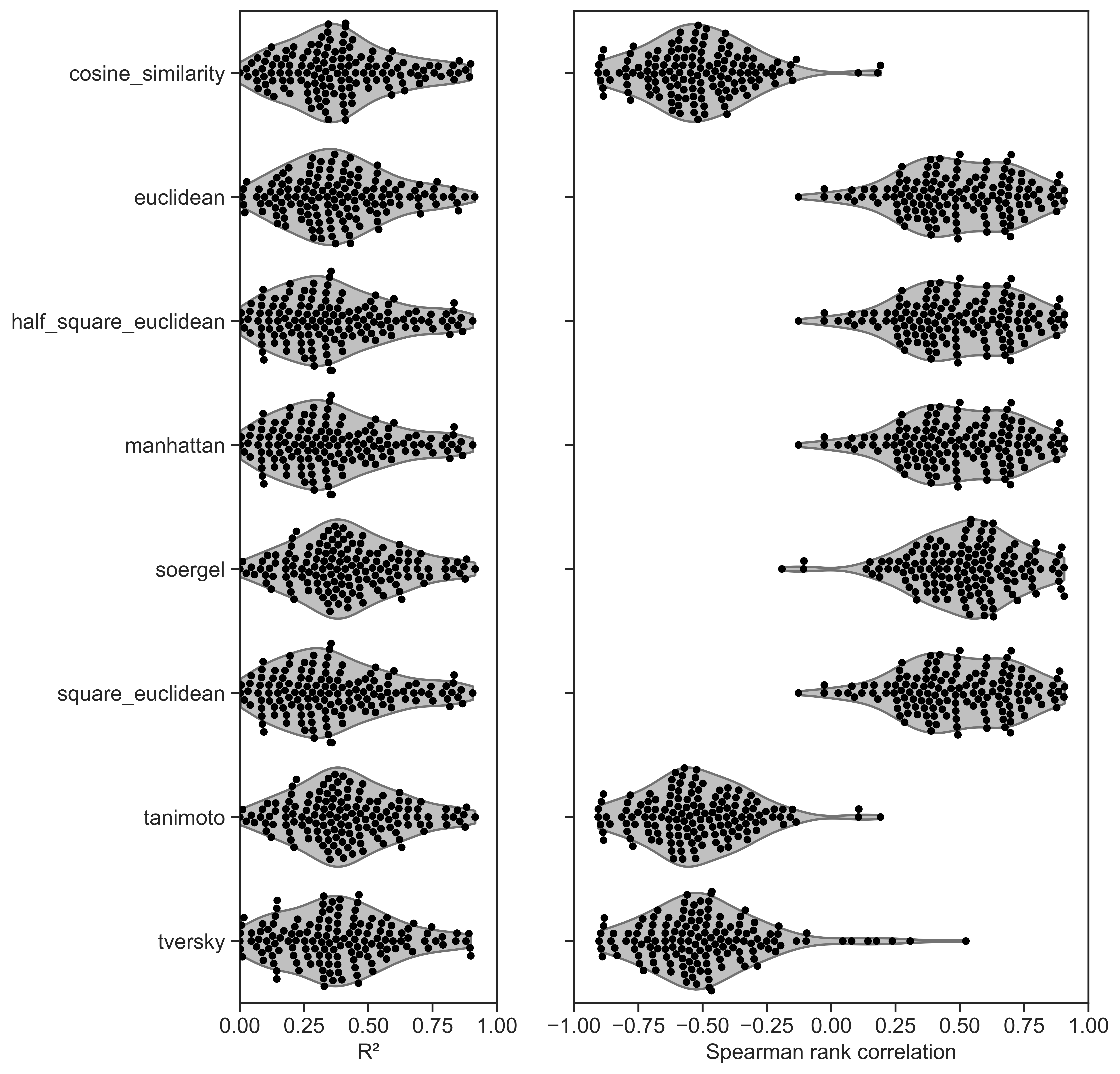

Supplement: S12 Fig — Distribution of R2 values (left) and Spearman rank correlation values (right) between RMSD and various metrics of SIFts similarities (y axis), calculated for a redocking experiment of 144 RNA-ligand complexes. (PNG) [file pcbi.1009783.s013.png]

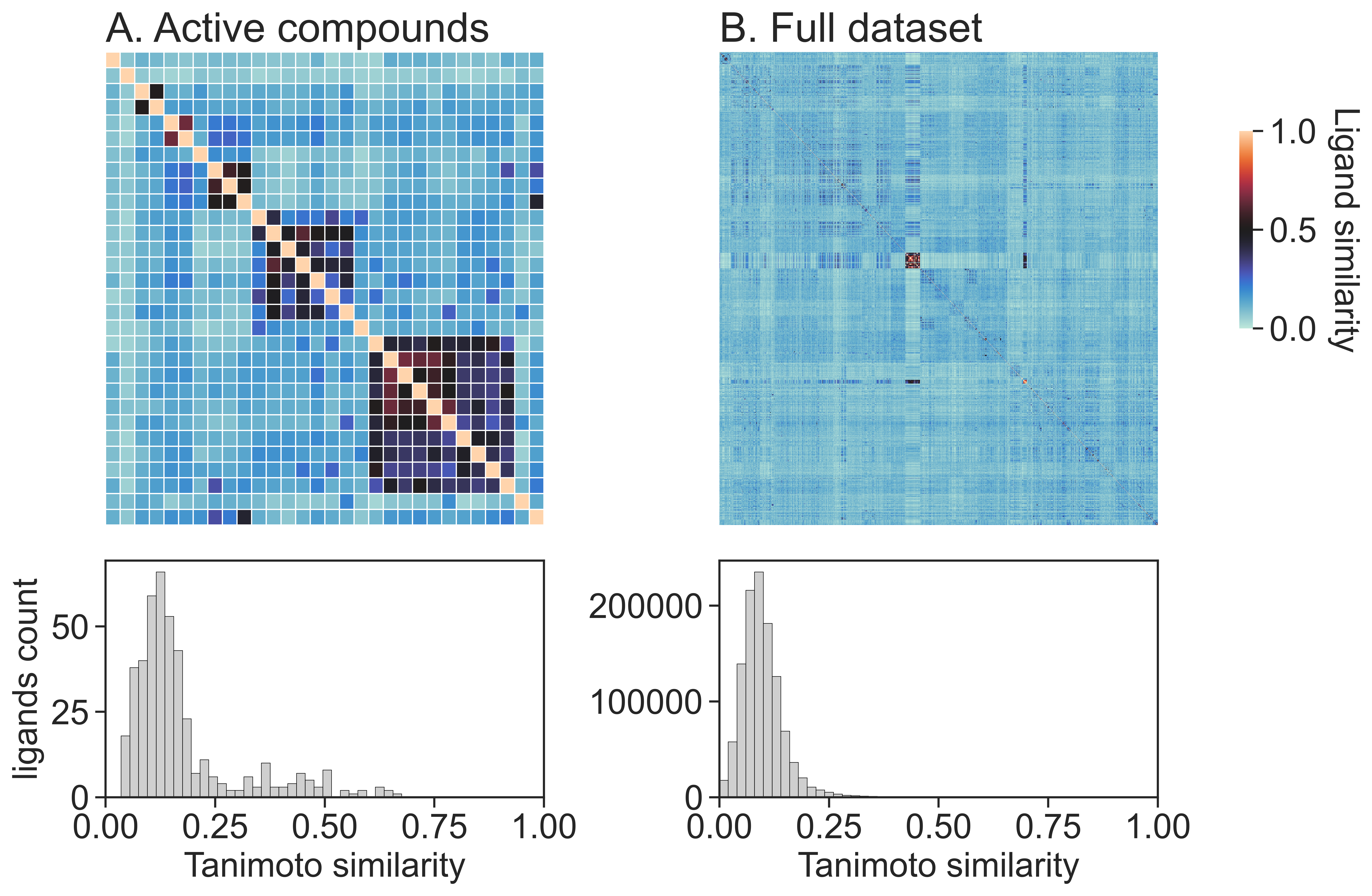

Supplement: S13 Fig — The heatmaps (upper row) and the histogram (lower row) of similarity of all ligand pairs in the HIV-1 dataset expressed as Tanimoto coefficient (ranging from 0 to 1, the higher the value, the more similar are the ligands) (A) for the subset of active compounds and (B) the complete dataset. (PNG) [file pcbi.1009783.s014.png]

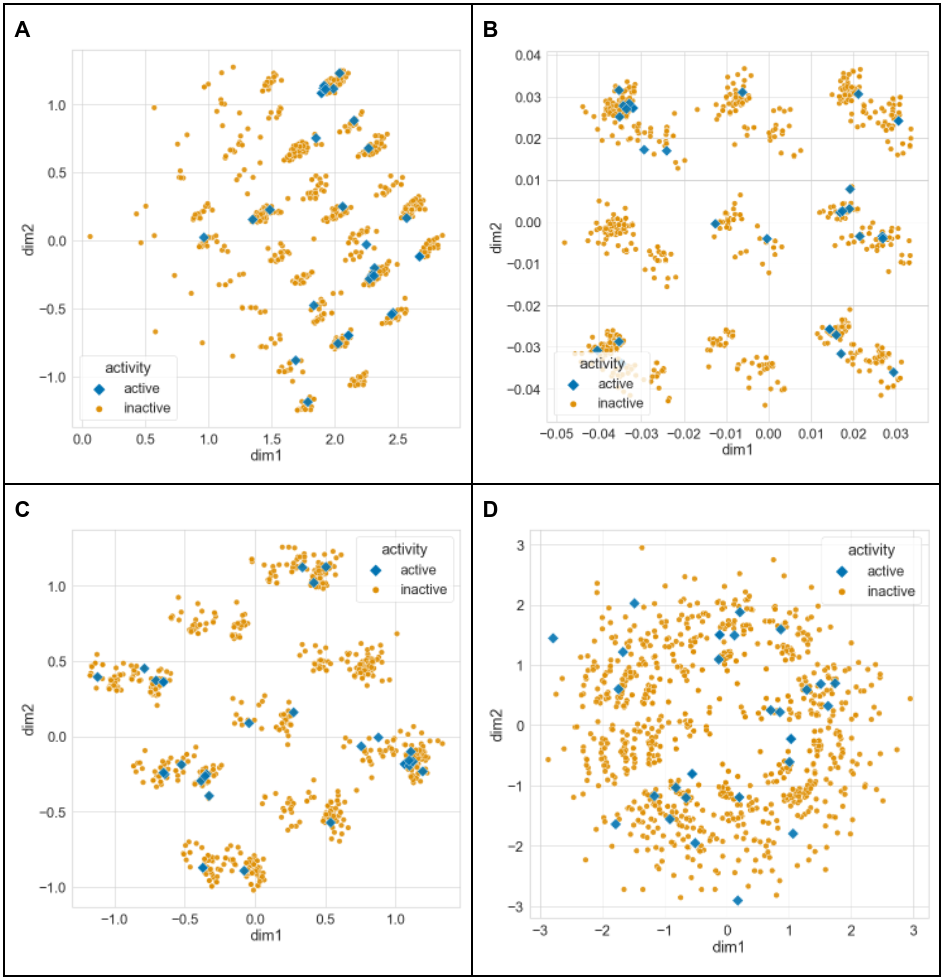

Supplement: S14 Fig — Data was mapped using (A) TruncatedSVD, (B) FastICA, (C) KernelPCA, and (D) MDS. (PNG) [file pcbi.1009783.s015.png]

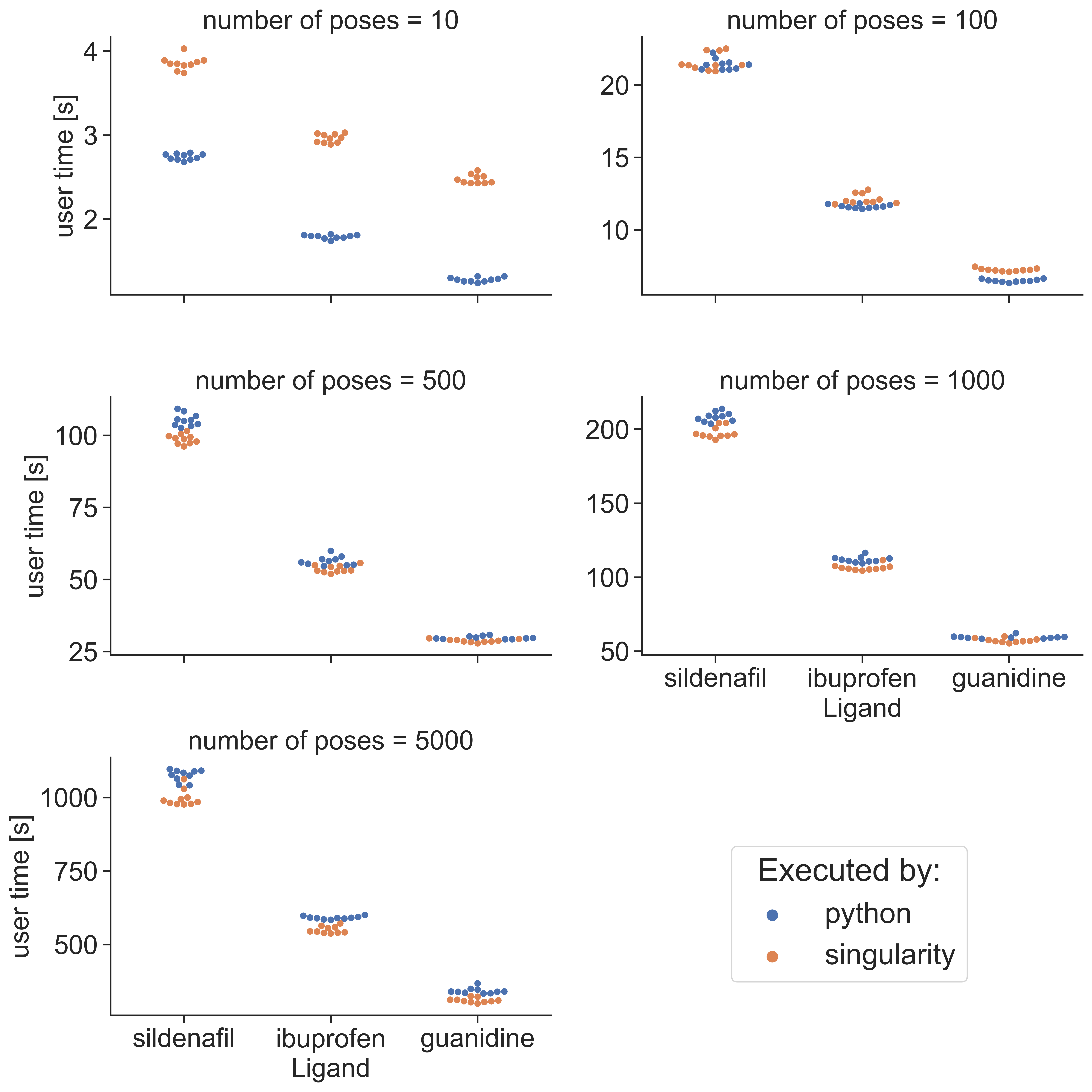

Supplement: S15 Fig — The fingeRNAt executed as a python script and as a singularity image. The number of poses analyzed was 10, 100, 500, 1000, and 5000 poses. The benchmark was performed on Ubuntu Linux 20.04 with Intel(R) Core(TM) i5-8400 CPU and 32 GB RAM. (PNG) [file pcbi.1009783.s016.png]

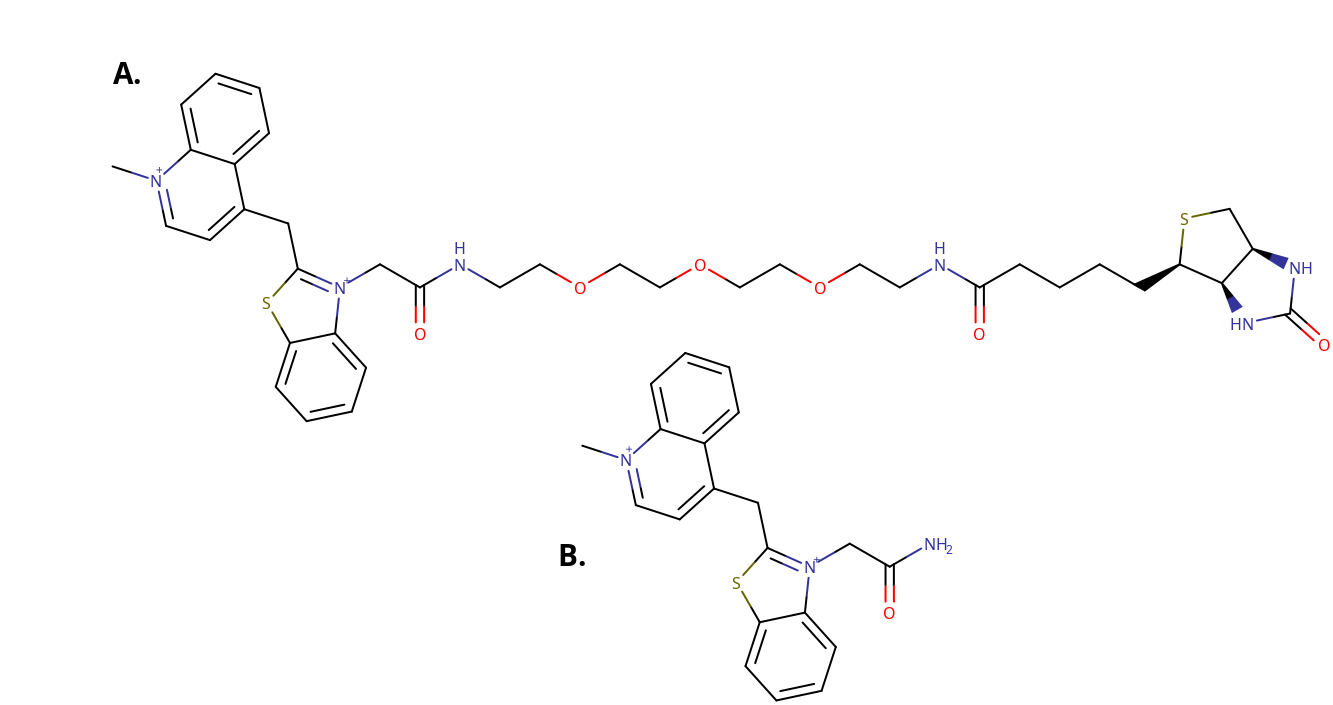

Supplement: S16 Fig — (A) Structure of TO1-Biotin solved in complex with Mango-III (A10U) aptamer (6E8U), and (B) structure of TO1 N-acetamide used for RMSD calculation for submitted models. (PNG) [file pcbi.1009783.s017.png]
