# Supplementary material for: fingeRNAt—A novel tool for high-throughput analysis of nucleic acid-ligand interactions
Source: PLoS Comput Biol. 2022 Jun 2;18(6):e1009783. doi: 10.1371/journal.pcbi.1009783 (PMC9197077; doi:10.1371/journal.pcbi.1009783)
Supplement: S1 Table — (PDF) [file pcbi.1009783.s018.pdf]

S1 Table. Comparison of the features of the fingeRNAt software (this manuscript) and similar programs (Arpeggio, PLIP2021, and ProLIF).

	fingeRNAt	Arpeggio	PLIP2021	ProLIF
**Receptor**				
Nucleic acids	Yes	Yes	Yes	Yes
Proteins	No	Yes	Yes	Yes
**Ligands**				
RNA	Yes	Yes/No*	Yes	Yes
DNA	Yes	Yes/No*	Yes	Yes
LNA (locked nucleic acid)	Yes	Yes/No**	Yes	Yes
Protein	Yes	Yes	Yes	Yes
Small molecule	Yes	Yes/No***	Yes	Yes
Ions	Yes	Yes	Yes	No
Detection of interactions	Yes	Yes	Yes	Yes
Fingerprints	Yes<br>For residues<br>Three levels of resolution	Yes<br>For individual atoms	No	Yes
Similarity/distance matrix	Yes	No	No	Yes
Number of metrics available	8	n/a	n/a	1
**Non-covalent bonds detected**				
Any contact	Yes (plugin)	Partially⁺	No	Yes (plugin)
Hydrogen Bond	Yes	Yes	Yes	Yes
Weak Hydrogen Bond	Yes (plugin)	Yes	No	No
Halogen Bond	Yes	Yes	Yes	Yes

Ionic	Yes	Yes	Yes	Yes
Metal Complex[ɤ]	Yes	Yes	Yes	Yes
Mg^{2+} specific	Yes	No	No	No
K^+ specific	Yes	No	No	No
Na^+ specific	Yes	No	No	No
Other	Yes	No	No	No
Aromatic	Yes	Yes	Yes	Yes
Pi-cation	Yes	No	Yes	Yes
Pi-anion	Yes	No	No	No
Hydrophobic	Yes	Yes	Yes	Yes
Carbonyl-carbonyl	Yes (plugin)	Yes	No	No
Polar	Yes (plugin)	Yes	No	No
Weak Polar	Yes (plugin)	Yes	No	No
Nucleic acid - amino acid	Yes (plugin)	No	No	No
Water-mediated	Yes	Yes	Yes	No
Ion interaction profiles	Yes	No	No	No
Multipolar halogen interactions	Yes (plugin)	No	No	No
User defined interactions	Yes (plugin)	No	No	Yes (python encoded)
**Software features**				
Customization of detection thresholds	Yes	No	Yes	Yes
Plugins for detecting custom interactions	Yes	No	No	Yes (python encoded)
Recent software update[†]	Dec 21, 2021	Jun 22, 2018	May 17, 2021	Aug 25, 2021

Availability<br>Command line<br>GUI<br>Web server<br>Docker<br>Singularity<br>Conda env.<br>Python module	 Yes<br>Yes<br>No<br>No<br>Yes<br>Yes<br>No	 Yes<br>No<br>Yes<br>Yes<br>No<br>Yes<br>No	 Yes<br>No<br>Yes<br>Yes<br>Yes<br>No<br>Yes	 No<br>No<br>No<br>No<br>No<br>Yes<br>Yes
License	GPL-3.0 License	GPL-3.0 License	GPL-2.0 License	Apache License, Version 2.0
Source code	https://github.com/n-szulc/fingeRNAt/	https://github.com/harryjubb/arpeggio	https://github.com/pharmai/plip	https://github.com/chemosim-lab/ProLIF

* No, if the receptor is RNA/DNA

** Works for protein as a receptor, not for nucleic acid as a receptor

*** No if the receptor is RNA (works unstable, e.g., works for 1AJU, but fails to run on 1Q8N or 5SWE) or DNA (does not work for 3EM2 or 3MVB)

[+] Hardcoded for atoms in Van der Waals interaction distance and within 5 Å of another atom

[ɣ] Any metal

[†] As of 2021-12-21
