# Supplementary material for: fingeRNAt—A novel tool for high-throughput analysis of nucleic acid-ligand interactions
Source: PLoS Comput Biol. 2022 Jun 2;18(6):e1009783. doi: 10.1371/journal.pcbi.1009783 (PMC9197077; doi:10.1371/journal.pcbi.1009783)
Supplement: S2 Table — (PDF) [file pcbi.1009783.s019.pdf]

S2 Table. The total number of interactions detected for RNA-ligand complexes and the number of RNA-ligand complexes with at least one occurrence of a given interaction.

Interaction	all interactions detected		complexes with this interaction	
	count	percentage of all detected interactions	count	percentage of all complexes
**Hydrogen bond**	5026	50.09%	206	99.52%
**Lipophilic**	3582	35.70%	196	94.69%
**Cation-anion**	899	8.96%	127	61.35%
**Water-mediated**	151	1.50%	31	14.98%
**Pi-stacking**	146	1.46%	62	29.95%
**Pi-cation**	96	0.96%	66	31.88%
**Ion-mediated (K ion)**	64	0.64%	13	6.28%
**Ion-mediated (Mg ion)**	36	0.36%	16	7.73%
**Pi-anion**	28	0.28%	22	10.63%
**Halogen bond**	6	0.06%	4	1.93%
