# Supplementary material for: fingeRNAt—A novel tool for high-throughput analysis of nucleic acid-ligand interactions
Source: PLoS Comput Biol. 2022 Jun 2;18(6):e1009783. doi: 10.1371/journal.pcbi.1009783 (PMC9197077; doi:10.1371/journal.pcbi.1009783)
Supplement: S3 Table — (A) Total number of interactions detected for RNA-ligand complexes; (B) the number and (C) the percentage of RNA-ligand complexes with at least one occurrence of a given interaction. (PDF) [file pcbi.1009783.s020.pdf]

**S3 Table. Statistics of hydrogen bonds detected in macromolecular complexes using various methods - without (the default method) and with taking the position of hydrogen atoms into account (hydrogens added with OpenBabel, RDKit, or PyMOL).** (A) Total number of interactions detected for RNA-ligand complexes; (B) the number and (C) the percentage of RNA-ligand complexes with at least one occurrence of a given interaction.

| hydrogen bond detection method | (A) Number of interactions | (B) Structures with this interaction | (C) % of structures with this interaction |
|---|---|---|---|
| **No H (default)** | 5026 | 206 | 99.52 |
| **With H: OpenBabel** | 1735 | 198 | 95.65 |
| **With H: RDKit** | 1746 | 199 | 96.14 |
| **With H: PyMOL** | 1735 | 195 | 94.20 |
| **With H: Chimera** | 1822 | 201 | 97.10 |
