# Supplementary material for: fingeRNAt—A novel tool for high-throughput analysis of nucleic acid-ligand interactions
Source: PLoS Comput Biol. 2022 Jun 2;18(6):e1009783. doi: 10.1371/journal.pcbi.1009783 (PMC9197077; doi:10.1371/journal.pcbi.1009783)
Supplement: S4 Table — (PDF) [file pcbi.1009783.s021.pdf]

S4 Table. Statistics of complexes and detected Pi-stacking interactions in the RNA-ligand dataset for ligands with or without at least one aromatic ring.

ligand with aromatic ring(s)	complexes with this kind of ligand		complexes in which Pi-stacking interaction is present	
**no**	129	62.32%	0	0.00%
**yes**	78	37.68%	62	79.49%
