# Supplementary material for: fingeRNAt—A novel tool for high-throughput analysis of nucleic acid-ligand interactions
Source: PLoS Comput Biol. 2022 Jun 2;18(6):e1009783. doi: 10.1371/journal.pcbi.1009783 (PMC9197077; doi:10.1371/journal.pcbi.1009783)
Supplement: S5 Table — (PDF) [file pcbi.1009783.s022.pdf]

S5 Table. Statistics of complexes and detected hydrogen bonds in the RNA-ligand dataset for ligands with or without at least one hydrogen bond donor and acceptor.

**ligand with hydrogen bond acceptor**	**ligand with hydrogen bond donor**	**complexes with this kind of ligand**		**complexes in which hydrogen bond is present**	
**no**	**yes**	7	3.38%	7	100.00%
**yes**	**no**	1	0.48%	0	0.00%
	**yes**	199	96.14%	199	100.00%
