# Supplementary material for: fingeRNAt—A novel tool for high-throughput analysis of nucleic acid-ligand interactions
Source: PLoS Comput Biol. 2022 Jun 2;18(6):e1009783. doi: 10.1371/journal.pcbi.1009783 (PMC9197077; doi:10.1371/journal.pcbi.1009783)
Supplement: S6 Table — (PDF) [file pcbi.1009783.s023.pdf]

S6 Table. Statistics of complexes and detected cation-anion interactions in the RNA-ligand dataset for ligands with or without at least one charged atom.

ligand	complexes with this kind of ligand		complexes in which cation-anion interaction is present	
**neutral**	24	11.59%	0	0.00%
**charged**	183	88.41%	127	69.40%
