# Supplementary material for: fingeRNAt—A novel tool for high-throughput analysis of nucleic acid-ligand interactions
Source: PLoS Comput Biol. 2022 Jun 2;18(6):e1009783. doi: 10.1371/journal.pcbi.1009783 (PMC9197077; doi:10.1371/journal.pcbi.1009783)
Supplement: S7 Table — (PDF) [file pcbi.1009783.s024.pdf]

S7 Table. **Statistics of the lengths for observed non-covalent interactions in a dataset of experimentally solved RNA-ligand structures.**

Interaction	bond length, Å					
	mean	std	min	median	max	detection threshold
**Hydrogen bond**	3.27	0.42	1.20	3.31	3.90	3.9
**Lipophilic**	3.53	0.54	1.33	3.69	4.00	4.0
**Cation-anion**	4.32	0.89	2.43	4.60	5.50	5.5
**Water-mediated: ligand-water**	2.84	0.22	2.03	2.86	3.40	3.5
**Water-mediated: water-RNA**	2.98	0.27	2.30	2.95	3.48	3.48
**Pi-stacking**	3.87	0.41	3.29	3.77	5.42	5.5
**Pi-cation**	4.78	0.69	3.04	4.78	5.99	6.0
**Ion-mediated (K ion): ion-RNA**	3.17	0.31	2.43	3.12	3.89	3.9
**Ion-mediated (K ion): ligand-ion**	3.00	0.28	2.74	2.85	3.58	3.58
**Ion-mediated (Mg ion): ion-RNA**	2.54	0.40	1.58	2.55	3.18	3.2
**Ion-mediated (Mg ion): ligand-ion**	2.29	0.34	1.58	2.16	3.00	3.00
**Pi-anion**	4.39	0.60	3.20	4.37	5.44	6.0
**Halogen bond**	3.62	0.51	2.63	3.81	3.97	4.0
