# Supplementary material for: fingeRNAt—A novel tool for high-throughput analysis of nucleic acid-ligand interactions
Source: PLoS Comput Biol. 2022 Jun 2;18(6):e1009783. doi: 10.1371/journal.pcbi.1009783 (PMC9197077; doi:10.1371/journal.pcbi.1009783)
Supplement: S8 Table — (PDF) [file pcbi.1009783.s025.pdf]

S8 Table. Statistics of hydrogen bonds formed by different RNA atoms.

Atom	Interaction count	% of all interactions	
**OP1**	451	8.97%	23.46%
**OP2**	728	14.48%	
**O2'**	261	5.19%	15.68%
**O3'**	111	2.21%	
**O4'**	199	3.96%	
**O5'**	217	4.32%	
**N1**	273	5.43%	60.86%
**N2**	156	3.10%	
**N3**	486	9.67%	
**N4**	261	5.19%	
**N6**	203	4.04%	
**N7**	656	13.05%	
**O2**	268	5.33%	
**O4**	388	7.72%	
**O6**	368	7.32%	
