# Supplementary material for: fingeRNAt—A novel tool for high-throughput analysis of nucleic acid-ligand interactions
Source: PLoS Comput Biol. 2022 Jun 2;18(6):e1009783. doi: 10.1371/journal.pcbi.1009783 (PMC9197077; doi:10.1371/journal.pcbi.1009783)
Supplement: S9 Table — (PDF) [file pcbi.1009783.s026.pdf]

S9 Table. Total number of hydrogen bonds formed by the nucleotides and the percentage of all hydrogen bonds formed by the given nucleotide using a given face of the nucleobase (H = Hoogsteen, WC = Watson-Crick, S = sugar).

nucleobase	total interaction count	face	% of all interactions
A	1089	H	22.4%
		WC	31.2%
		S	8.9%
G	2306	H	41.3%
		WC	23.4%
		S	12.6%
C	772	H	31.0%
		WC	63.0%
		S	19.3%
U	859	H	45.6%
		WC	68.8%
		S	13.8%
