# Supplementary material for: fingeRNAt—A novel tool for high-throughput analysis of nucleic acid-ligand interactions
Source: PLoS Comput Biol. 2022 Jun 2;18(6):e1009783. doi: 10.1371/journal.pcbi.1009783 (PMC9197077; doi:10.1371/journal.pcbi.1009783)
Supplement: S10 Table — (PDF) [file pcbi.1009783.s027.pdf]

S10 Table. Statistics of lipophilic interactions formed by different RNA atoms.

Atom	Interaction count	% of all interactions	
**C1'**	294	8.21%	25.96%
**C2'**	215	6.00%	
**C3'**	105	2.93%	
**C4'**	181	5.05%	
**C5'**	135	3.77%	
**C2**	786	21.94%	74.04%
**C4**	519	14.49%	
**C5**	581	16.22%	
**C6**	590	16.47%	
**C8**	176	4.91%	
