# Supplementary material for: fingeRNAt—A novel tool for high-throughput analysis of nucleic acid-ligand interactions
Source: PLoS Comput Biol. 2022 Jun 2;18(6):e1009783. doi: 10.1371/journal.pcbi.1009783 (PMC9197077; doi:10.1371/journal.pcbi.1009783)
Supplement: S11 Table — (PDF) [file pcbi.1009783.s028.pdf]

S11 Table. **Statistics of Pi-anion interactions formed by different RNA groups (when RNA is an anion acceptor) and atoms (where RNA is an anion donor).**

RNA role	Group	Interaction count	% of all interactions	
**RNA as anion acceptor**	**5-atoms ring**	10	35.71%	82.14%
	**6-atom rings**	13	46.43%	
**RNA as anion donor**	**OP1**	4	14.29%	17.86%
	**OP2**	1	3.57%	
