# Supplementary material for: fingeRNAt—A novel tool for high-throughput analysis of nucleic acid-ligand interactions
Source: PLoS Comput Biol. 2022 Jun 2;18(6):e1009783. doi: 10.1371/journal.pcbi.1009783 (PMC9197077; doi:10.1371/journal.pcbi.1009783)
Supplement: S12 Table — (PDF) [file pcbi.1009783.s029.pdf]

S12 Table. Statistics of halogen bonds formed by different RNA atoms.

Atom	Interaction count	% of all interactions	
**N3**	1	16.67%	16.67%
**O2'**	3	50.00%	83.33%
**O3'**	1	16.67%	
**O4'**	1	16.67%	
