# Supplementary material for: fingeRNAt—A novel tool for high-throughput analysis of nucleic acid-ligand interactions
Source: PLoS Comput Biol. 2022 Jun 2;18(6):e1009783. doi: 10.1371/journal.pcbi.1009783 (PMC9197077; doi:10.1371/journal.pcbi.1009783)
Supplement: S13 Table — (PDF) [file pcbi.1009783.s030.pdf]

S13 Table. Statistics of all types of interactions formed by different RNA atoms and groups.

atom / group	Interaction count	% of all interactions	
**OP1**	844	8.41%	21.63%
**OP2**	1328	13.23%	
**O2'**	293	2.92%	17.84%
**O3'**	117	1.17%	
**O4'**	227	2.26%	
**O5'**	224	2.23%	
**C1'**	294	2.93%	
**C2'**	215	2.14%	
**C3'**	105	1.05%	
**C4'**	181	1.80%	
**C5'**	135	1.34%	
**N1**	275	2.74%	60.53%
**N2**	158	1.57%	
**N3**	508	5.06%	
**N4**	268	2.67%	
**N6**	212	2.11%	
**N7**	673	6.70%	
**O2**	275	2.74%	
**O4**	390	3.88%	
**O6**	402	4.00%	
**C2**	786	7.83%	
**C4**	519	5.17%	
**C5**	581	5.79%	
**C6**	590	5.88%	
**C8**	176	1.75%	
**C4,C5,N7,C8,N9**	79	0.79%	
**N1,C2,N3,C4,C5,C6**	186	1.85%	
