# Supplementary material for: fingeRNAt—A novel tool for high-throughput analysis of nucleic acid-ligand interactions
Source: PLoS Comput Biol. 2022 Jun 2;18(6):e1009783. doi: 10.1371/journal.pcbi.1009783 (PMC9197077; doi:10.1371/journal.pcbi.1009783)
Supplement: S14 Table — (PDF) [file pcbi.1009783.s031.pdf]

S14 Table. Linear least-squares regression R^2 values calculated for parameters of structures from the RNA-Puzzles collective experiment and redocking experiment.

Structures	Parameter	Parameter/Tversky fingerprint similarity R^2
RNA Puzzle 23	RMSD (RNA)	0.61
	INF all (RNA)	0.44
	RMSD (Ligand)	0.37
Redocking	RMSD (Ligand)	0.60
