# Supplementary material for: fingeRNAt—A novel tool for high-throughput analysis of nucleic acid-ligand interactions
Source: PLoS Comput Biol. 2022 Jun 2;18(6):e1009783. doi: 10.1371/journal.pcbi.1009783 (PMC9197077; doi:10.1371/journal.pcbi.1009783)
Supplement: S15 Table — The three best values in a given category are highlighted in green. Due to errors in the structure of the ligand, it was not possible to calculate ligand RMSD for models submitted by the Xiao group. (PDF) [file pcbi.1009783.s032.pdf]

S15 Table. **Quality of models submitted to the RNA-Puzzles competition round 23.** The three best values in a given category are highlighted in green. Due to errors in the structure of the ligand, it was not possible to calculate ligand RMSD for models submitted by the Xiao group.

Molecule	RNA			Ligand	Interaction fingerprints			
	RMSD	DI all	INF all	RMSD	Tversky	Tanimoto	Euclidean	Manhattan
23_solution_0	0.00	0.00	1.00	0.00	1.00	1.00	0.01	0.00
Bujnicki_01	12.16	386.74	0.03	17.01	0.00	0.00	5.00	25.00
Bujnicki_02	12.17	175.02	0.07	20.61	0.07	0.03	5.39	29.00
Bujnicki_03	11.95	353.33	0.03	12.48	0.07	0.04	5.20	27.00
Chen_01	11.42	24.69	0.46	10.34	0.20	0.14	4.36	19.00
Chen_02	13.02	22.25	0.59	7.24	0.27	0.20	4.00	16.00
Chen_03	10.59	18.75	0.57	7.92	0.27	0.17	4.47	20.00
Das_01	11.16	22.57	0.50	9.09	0.20	0.11	4.90	24.00
Das_02	12.83	20.64	0.62	7.42	0.27	0.15	4.69	22.00
Das_03	12.55	21.85	0.58	6.35	0.53	0.32	4.12	17.00
Das_04	12.05	21.31	0.57	6.79	0.47	0.30	4.00	16.00
Das_05	12.05	20.30	0.59	7.34	0.40	0.25	4.24	18.00
Das_06	9.69	15.17	0.64	3.59	0.20	0.10	5.10	26.00
Das_07	8.13	14.79	0.55	3.19	0.47	0.33	3.74	14.00
Das_08	10.68	20.89	0.51	5.24	0.40	0.23	4.47	20.00
Das_09	11.78	21.20	0.56	7.26	0.27	0.16	4.58	21.00
Das_10	12.32	24.37	0.51	9.25	0.20	0.12	4.80	23.00
Ding_01	11.27	19.35	0.58	6.53	0.33	0.17	4.90	24.00
Ding_02	11.68	24.21	0.48	6.64	0.27	0.13	5.29	28.00
Ding_03	11.46	20.60	0.56	6.78	0.27	0.15	4.69	22.00
Ding_04	12.75	24.07	0.53	7.48	0.13	0.09	4.58	21.00
Ding_05	13.26	24.63	0.54	7.76	0.20	0.12	4.80	23.00
Dokholyan_01	12.43	32.29	0.39	6.84	0.07	0.03	5.29	28.00
RNAComposerHuman_01	13.10	23.17	0.57	6.45	0.00	0.00	4.36	19.00
RNAComposerHuman_02	13.23	23.74	0.56	6.47	0.00	0.00	4.36	19.00
RNAComposerHuman_03	14.21	27.78	0.51	6.60	0.07	0.06	4.12	17.00
Xiao_01	10.76	25.00	0.43	-	0.00	0.00	5.10	26.00
Xiao_02	10.90	23.02	0.47	-	0.00	0.00	4.47	20.00
Xiao_03	10.89	24.29	0.45	-	0.00	0.00	4.90	24.00
Xiao_04	18.22	59.36	0.31	-	0.27	0.24	3.61	13.00
Xiao_05	17.20	61.82	0.28	-	0.00	0.00	4.36	19.00
