# Supplementary material for: fingeRNAt—A novel tool for high-throughput analysis of nucleic acid-ligand interactions
Source: PLoS Comput Biol. 2022 Jun 2;18(6):e1009783. doi: 10.1371/journal.pcbi.1009783 (PMC9197077; doi:10.1371/journal.pcbi.1009783)
Supplement: S16 Table — Statistics of relationship between RMSD and SIFts similarity—(A) Tanimoto coefficient and (B) Tversky distance, calculated for a redocking experiment of 144 RNA-ligand complexes. (PDF) [file pcbi.1009783.s033.pdf]

S16 Table. Statistics of relationship between RMSD and SIFts similarity - (A) Tanimoto coefficient and (B) Tversky distance, calculated for a redocking experiment of 144 RNA-ligand complexes.

	Spearman	Pearson	Kendall	R^2
**A. RMSD vs. Tanimoto coefficient**				
**mean**	-0.523	-0.617	-0.400	0.417
**std**	0.205	0.192	0.169	0.206
**min**	-0.906	-0.957	-0.802	0.000
**0.250**	-0.639	-0.740	-0.503	0.282
**0.500**	-0.535	-0.630	-0.402	0.397
**0.750**	-0.401	-0.531	-0.293	0.548
**max**	0.192	0.212	0.145	0.916
**B. RMSD vs. Tversky**				
**mean**	-0.491	-0.564	-0.391	0.380
**std**	0.242	0.249	0.203	0.216
**min**	-0.903	-0.948	-0.797	0.000
**0.250**	-0.641	-0.716	-0.515	0.220
**0.500**	-0.516	-0.605	-0.399	0.366
**0.750**	-0.375	-0.465	-0.287	0.513
**max**	0.524	0.600	0.425	0.899
