# Supplementary material for: fingeRNAt—A novel tool for high-throughput analysis of nucleic acid-ligand interactions
Source: PLoS Comput Biol. 2022 Jun 2;18(6):e1009783. doi: 10.1371/journal.pcbi.1009783 (PMC9197077; doi:10.1371/journal.pcbi.1009783)
Supplement: S17 Table — (PDF) [file pcbi.1009783.s034.pdf]

S17 Table. Silhouette score, Calinski-Harabasz score, and Davies-Bouldin score calculated for a various number of clusters, for all-interactions dataset and the one with lipophilic interactions removed.

clusters	All-interactions dataset			Lipophilic interactions removed		
	silhouette score	Calinski-Harabasz score	Davies-Bouldin score	silhouette score	Calinski-Harabasz score	Davies-Bouldin score
2	0.39	1,067.90	1.07	0.44	1,198.77	1.03
3	0.48	1,751.58	0.72	0.51	1,834.79	0.68
4	0.50	2,096.95	0.68	0.55	2,432.19	0.63
5	0.46	2,085.33	0.76	0.58	2,608.03	0.62
6	0.45	2,023.32	0.80	0.60	3,102.29	0.67
7	0.46	2,016.26	0.79	0.65	3,680.02	0.59
8	0.46	2,126.40	0.79	0.68	4,568.15	0.53
9	0.48	2,232.21	0.72	**0.72**	5,959.66	0.40
10	0.48	2,361.72	0.70	0.71	6,536.59	0.41
11	0.49	2,455.81	0.70	0.70	7,135.19	0.41
12	0.49	2,522.17	0.71	0.71	7,663.26	0.41
13	0.51	2,675.70	0.70	0.69	7,846.86	0.45
14	**0.52**	2,734.86	0.70	0.70	8,088.73	0.42
15	0.52	2,801.87	0.68	0.68	8,620.76	0.48
20	0.52	3,060.47	0.66	0.70	11,452.47	0.46
30	0.45	3,153.28	0.77	0.60	11,364.59	0.62
