# Supplementary material for: fingeRNAt—A novel tool for high-throughput analysis of nucleic acid-ligand interactions
Source: PLoS Comput Biol. 2022 Jun 2;18(6):e1009783. doi: 10.1371/journal.pcbi.1009783 (PMC9197077; doi:10.1371/journal.pcbi.1009783)
Supplement: S18 Table — Two-tailed p-values obtained in a t-test for the means of two independent samples of scores are reported; p-values ≤ 0.05 are bolded. (PDF) [file pcbi.1009783.s035.pdf]

S18 Table. **Composition of the input dataset and clusters (number of active and inactive compounds, and percentage of active compounds in a given cluster), and *p*-values for comparing the ratio of active compounds in the input dataset and in a given cluster.** Two-tailed *p*-values obtained in a *t*-test for the means of two independent samples of scores are reported; *p*-values ≤ 0.05 are bolded.

**A. All-interactions dataset**					**B. Lipophilic interactions removed**				
**cluster**	**active cpds count**	**inactive cpds count**	**% of active cpds**	***p*-value**	**cluster**	**active cpds count**	**inactive cpds count**	**% of active cpds**	***p*-value**
Input dataset	30	1478	2.03	-	Input dataset	30	1478	2.03	-
0	0	69	0.00	0.83	0	9	181	4.97	0.08
1	2	83	2.41	0.84	1	0	80	0.00	**0.00**
2	1	83	1.20	0.71	2	3	301	1.00	0.14
3	2	172	1.16	0.06	3	5	290	1.72	0.72
4	2	69	2.90	0.88	4	3	155	1.94	0.94
5	1	158	0.63	0.34	5	7	166	4.22	0.18
6	1	106	0.94	0.19	6	0	130	0.00	**0.00**
7	8	120	6.67	0.29	7	1	113	0.88	0.24
8	**3**	126	2.38	**0.05**	8	2	62	3.23	0.61
9	0	52	0.00	**0.00**					
10	0	92	0.00	**0.00**					
11	**6**	133	4.51	**0.00**					
12	**3**	75	4.00	**0.00**					
13	1	58	1.72	0.76					
14	0	82	0.00	0.68					
