# Supplementary material for: fingeRNAt—A novel tool for high-throughput analysis of nucleic acid-ligand interactions
Source: PLoS Comput Biol. 2022 Jun 2;18(6):e1009783. doi: 10.1371/journal.pcbi.1009783 (PMC9197077; doi:10.1371/journal.pcbi.1009783)
Supplement: S19 Table — The fingeRNAt executed as a python script or as a singularity image. The number of poses analyzed was 10, 100, 500, 1000, and 5000 poses. The benchmark was performed on Ubuntu Linux 20.04 with Intel(R) Core(TM) i5-8400 CPU and 32 GB RAM. (PDF) [file pcbi.1009783.s036.pdf]

S19 Table. **Mean FULL fingerprint calculation times (in seconds, the average from 10 experiments) of docking poses of small molecule ligands of various sizes (guanidine, ibuprofen, and sildenafil) to guanidine III riboswitch (RNA with 39 residues).** The fingeRNAt executed as a python script or as a singularity image. The number of poses analyzed was 10, 100, 500, 1000, and 5000 poses. The benchmark was performed on Ubuntu Linux 20.04 with Intel(R) Core(TM) i5-8400 CPU and 32 GB RAM.

ligand	number of poses	Time [s]; fingeRNAt executed with:	
		python	singularity
guanidine	10	1.281	2.477
	100	6.501	7.249
	500	29.823	28.744
	1000	59.462	57.254
	5000	342.286	310.042
ibuprofen	10	1.791	2.962
	100	11.625	12.140
	500	56.455	53.642
	1000	111.915	106.434
	5000	591.041	549.643
sildenafil	10	2.742	3.855
	100	21.425	21.598
	500	105.369	98.756
	1000	208.389	197.745
	5000	1075.484	997.598
