# Supplementary material for: fingeRNAt—A novel tool for high-throughput analysis of nucleic acid-ligand interactions
Source: PLoS Comput Biol. 2022 Jun 2;18(6):e1009783. doi: 10.1371/journal.pcbi.1009783 (PMC9197077; doi:10.1371/journal.pcbi.1009783)
Supplement: S20 Table — (PDF) [file pcbi.1009783.s037.pdf]

S20 Table. Definitions of the criteria of nine non-covalent interactions calculated by the fingeRNAt.py.

Interaction	Criteria	Source
Hydrogen bond	Donor - acceptor distance < 3.9 Å<br>Also possible additional condition:<br>100° < donor - hydrogen - acceptor angle < 260°	[1-2]
Halogen bond	1. Halogen - acceptor distance < 4.0 Å<br>2. C-X-A angle ~ 165° ± 30°<br>3. X-A-A' angle ~ 120° ± 30°	[3]
Cation-anion	Cation - anion distance < 5.5 Å	[4]
Pi-cation/anion	1. Cation/anion - aromatic ring center distance < 6.0 Å<br>2. angle between the ring plane and the line between cation/anion - ring center ~ 90° ± 30°<br><br>In case of Pi - cation interaction, the aromatic ring is only from the nucleic acid side (as nucleic acids are negatively charged). However, Pi - anion interaction is considered both ways: (i) nucleic acid's aromatic ring - ligand's anion and (ii) nucleic acid's anion (from phosphate group; see above) - ligand's aromatic ring.	[5]
Pi-stacking: Sandwich & Parallel displaced	1. rings' centroids distance < 5.5 Å<br>2. rings' offset < 2.0 Å<br>3. angle between the ring planes < 30°	[6]
Pi-stacking: T-shaped	1. rings' centroids distance < 5.5 Å<br>2. rings' offset < 2.0 Å<br>3. angle between the ring planes ~ 90° ± 30°	[6]
Ion-mediated	1. ligand's nitrogen/oxygen/sulphur - ion distance <= X<br>2. nucleic acid's nitrogen/oxygen - ion distance <= X, where:<br>a) X = 3.2 Å for magnesium ion<br>b) X = 3.9 Å for potassium ion<br>c) X = 3.6 Å for sodium ion<br>d) X = 3.5 Å for other ions	[7-8]
Water-mediated	1. ligand's hydrogen bond donor/acceptor - water (oxygen) distance <= 3.5 Å	[9-10]

	2. nucleic acid's hydrogen bond donor/acceptor - water (oxygen) distance <= 3.5 Å	
Lipophilic	Nucleic acid's carbon - ligand's lipophilic atom distance <= 4.0 Å	[11]

References

1. Torshin IY, Weber IT, Harrison RW. Geometric criteria of hydrogen bonds in proteins and identification of `bifurcated' hydrogen bonds. Protein Eng Des Sel. 2002 May 1;15(5):359–63.
2. Adasme MF, Linnemann KL, Bolz SN, Kaiser F, Salentin S, Haupt VJ, et al. PLIP 2021: expanding the scope of the protein–ligand interaction profiler to DNA and RNA. Nucleic Acids Res. 2021 May 5;49(W1):W530–4.
3. Auffinger P, Hays FA, Westhof E, Ho PS. Halogen bonds in biological molecules. Proc Natl Acad Sci. 2004 Nov 30;101(48):16789–94.
4. Barlow DJ, Thornton JM. Ion-pairs in proteins. J Mol Biol. 1983 Aug 25;168(4):867–85.
5. Gallivan JP, Dougherty DA. Cation-pi interactions in structural biology. Proc Natl Acad Sci U S A. 1999 Aug 17;96(17):9459–64.
6. McGaughey GB, Gagné M, Rappé AK. pi-Stacking interactions. Alive and well in proteins. J Biol Chem. 1998 Jun 19;273(25):15458–63.
7. Zheng H, Chruszcz M, Lasota P, Lebioda L, Minor W. Data mining of metal ion environments present in protein structures. J Inorg Biochem. 2008 Sep 1;102(9):1765–76.
8. Zheng H, Shabalin IG, Handing KB, Bujnicki JM, Minor W. Magnesium-binding architectures in RNA crystal structures: validation, binding preferences, classification and motif detection. Nucleic Acids Res. 2015 Apr 20;43(7):3789–801.
9. Poornima CS, Dean PM. Hydration in drug design. 1. Multiple hydrogen-bonding features of water molecules in mediating protein-ligand interactions. J Comput Aided Mol Des. 1995 Dec;9(6):500–12.
10. Poornima CS, Dean PM. Hydration in drug design. 2. Influence of local site surface shape on water binding. J Comput Aided Mol Des. 1995 Dec 1;9(6):513–20.
11. Padroni G, N. Patwardhan N, Schapira M, E. Hargrove A. Systematic analysis of the interactions driving small molecule–RNA recognition. RSC Med Chem. 2020;11(7):802–13.
