# Supplementary material for: fingeRNAt—A novel tool for high-throughput analysis of nucleic acid-ligand interactions
Source: PLoS Comput Biol. 2022 Jun 2;18(6):e1009783. doi: 10.1371/journal.pcbi.1009783 (PMC9197077; doi:10.1371/journal.pcbi.1009783)
Supplement: S21 Table — (PDF) [file pcbi.1009783.s038.pdf]

S21 Table. Parameters accepted by the fingeRNAt.py.

Parameter	Status	Description
-r	Required	path to receptor file
-l	Optional	path to ligands file
-f	Optional	type of SIFt to be calculated; default: FULL
-addH	Optional	type of module to be used to add hydrogens to ligands' structures; default: OpenBabel
-o	Optional	path to save output
-custom	Optional	path to yaml file with information about additional interactions to be calculated
-fingerDISt	Optional	fingerDISt Distance Metrics to be calculated (fingerDISt will be directly run on the SIFts output file)
-dha	Optional	additional condition when detecting hydrogen bonds: Donor-Hydrogen-Acceptor angle
-h2o	Optional	Checks water-mediated interactions ; default: does not check
-print	Optional	prints detected interactions for each nucleic acid - ligand complex on screen
-detail	Optional	generates an additional file with detailed data on detected interactions
-wrapper	Optional	type of wrapper to post-process SIFt output
-verbose	Optional	provides additional information about performed calculations at the given moment
-debug	Optional	enters debug mode
-h; --help	Optional	shows help message
