# Supplementary material for: fingeRNAt—A novel tool for high-throughput analysis of nucleic acid-ligand interactions
Source: PLoS Comput Biol. 2022 Jun 2;18(6):e1009783. doi: 10.1371/journal.pcbi.1009783 (PMC9197077; doi:10.1371/journal.pcbi.1009783)
Supplement: S22 Table — (PDF) [file pcbi.1009783.s039.pdf]

S22 Table. Summary of detected chemical properties and external modules used in the fingeRNAt.py.

Properties	Input type	External module
Hydrogen bonds acceptors & donors	Receptor/Ligand	OpenBabel
Halogen bonds acceptors	Receptor	OpenBabel
Halogen bonds donors	Ligand	OpenBabel
Nucleic acid’s anions	Receptor	None; negative charges are arbitrary assigned to both oxygens (OP1 & OP2) of each residue's phosphate group
Cations & anions	Ligand	OpenBabel
Aromatic rings	Receptor	OpenBabel
Aromatic rings	Ligand	RDKit
Lipophilic atoms	Ligand	RDKit
