# Supplementary material for: fingeRNAt—A novel tool for high-throughput analysis of nucleic acid-ligand interactions
Source: PLoS Comput Biol. 2022 Jun 2;18(6):e1009783. doi: 10.1371/journal.pcbi.1009783 (PMC9197077; doi:10.1371/journal.pcbi.1009783)
Supplement: S23 Table — (PDF) [file pcbi.1009783.s040.pdf]

S23 Table. List of structures used to derive statistics on RNA-small molecule ligand interactions.

1AJU	1EI2	1FUF	1I9V	1JZY	1KOD	1N32	1NJN	1P9X	1TN2	1VQM	1YI2	2BE0	2ESI
2F4S	2FD0	2GIS	2HOJ	2O3W	2OGO	2UUC	2Z75	3C7R	3D2X	3DIY	3DW4	3FO6	3GCA
3GX5	3OWW	3SLM	3TZR	4V4H	4V5G	4V8A	1AKX	1ET4	1FYP	1IBL	1JZZ	1KQS	1N33
1NTA	1PBR	1TOB	1VQN	1YRJ	2BEE	2ESJ	2F4T	2G32	2GQ4	2HOM	2O3X	2PWT	2UXB
3B4A	3CC4	3DIG	3DJ0	3E5C	3G4M	3GER	3IQR	3SD3	3SLQ	3V7E	4V51	4V5Y	4V8C
1AM0	1EVV	1G4Q	1J5A	1K01	1LC4	1N8R	1NTB	1Q81	1UTS	1XBP	1YYK	2CKY	2ET3
2F4U	2G5K	2GUN	2JUK	2O3Y	2QWY	2UXD	3B4B	3CMA	3DIL	3DJ2	3E5E	3G6E	3GLP
3L3C	3SKI	3SUH	4FE5	4V52	4V64	4V8F	1ARJ	1F1T	1HNW	1J7T	1K73	1LVJ	1NBK
1NWY	1Q8N	1UUD	1XMQ	2A04	2DQQ	2ET4	2FCX	2G9C	2H0Z	2KXM	2OE5	2TOB	2VQE
3BNQ	3D0U	3DIO	3DLL	3E5F	3G71	3GOT	3LA5	3SKL	3SUX	4KQY	4V53	4V7T	1BYJ
1F27	1HNZ	1J8G	1KC8	1M90	1NEM	1O15	1QD3	1UUI	1XPF	2AU4	2EES	2ET5	2FCY
2GCV	2HHH	2NZ4	2OE8	2TRA	2W89	3C44	3D2G	3DIQ	3DS7	3EGZ	3G9C	3GX2	3MXH
3SKR	3T1Y	4LVV	4V57	4V7V	1EHT	1FMN	1I97	1JZX	1KOC	1MWL	1NJM	1O9M	1RAW
1VQ6	1Y26	2B57	2EEU	2ET8	2FCZ	2GDI	2HO6	2O3V	2OGN	2UUB	2Z74	3C5D	3D2V
3DIR	3DVV	3F2Q	3GAO	3GX3	3NPQ	3SKZ	3TD1	4P20	4V5C	4V85			
